# Supplementary material for: Safety and cost analysis of selective histopathological examination following appendicectomy and cholecystectomy (FANCY study): protocol and statistical analysis plan of a prospective observational multicentre study
Source: BMJ Open. 2019 Dec 23;9(12):e035912. doi: 10.1136/bmjopen-2019-035912 (PMC7008446; doi:10.1136/bmjopen-2019-035912)
Supplement: Supplementary data [file bmjopen-2019-035912supp001.pdf]

SUPPLEMENTARY MATERIAL 1

Questions of the scoring form 'Macroscopic examination of the appendix'

1) Who performed the macroscopic examination of the appendix?
Multiple answers possible
O Surgeon
O Surgical resident

2) Are there any macroscopic abnormalities suspicious for a malignant neoplasm during visual inspection and/or digital palpation?
O Yes (please specify below)
O No
O Proper assessment is not possible due to inflammation

2a. If yes, specify the macroscopic abnormalities
Multiple answers possible
O Visible tumour
O Palpable tumour
O Other, namely …

3) Do you believe additional histopathological examination by the pathologist is indicated?
O Yes, because of the above-mentioned abnormalities.
O Yes, because proper assessment of the appendix was not possible.
O Yes, because …
O No

Questions of the scoring form 'Macroscopic examination of the gallbladder'

1) Who performed the macroscopic examination of the gallbladder?
Multiple answers possible
O Surgeon
O Surgical resident

2) Are there any macroscopic abnormalities suspicious for a malignant neoplasm during visual inspection and/or digital palpation?
O Yes (please specify below)
O No

2a. If yes, specify the macroscopic abnormalities
Multiple answers possible

O	Ulcer	O	Wall thickening
O	Cyst	O	Polyp
O	Hardening	O	Tumour suspicious for malignancy
O	Irregularity	O	Other, namely …

3) Do you believe additional histopathological examination by the pathologist is indicated?
O Yes, because of the above-mentioned abnormalities.
O Yes, because …
O No
