# Supplementary material for: Safety and cost analysis of selective histopathological examination following appendicectomy and cholecystectomy (FANCY study): protocol and statistical analysis plan of a prospective observational multicentre study
Source: BMJ Open. 2019 Dec 23;9(12):e035912. doi: 10.1136/bmjopen-2019-035912 (PMC7008446; doi:10.1136/bmjopen-2019-035912)
Supplement: Author's manuscript [file bmjopen-2019-035912.draft_revisions.pdf]

BMJ Open is committed to open peer review. As part of this commitment we make the peer review history of every article we publish publicly available.

When an article is published we post the peer reviewers' comments and the authors' responses online. We also post the versions of the paper that were used during peer review. These are the versions that the peer review comments apply to.

The versions of the paper that follow are the versions that were submitted during the peer review process. They are not the versions of record or the final published versions. They should not be cited or distributed as the published version of this manuscript.

BMJ Open is an open access journal and the full, final, typeset and author-corrected version of record of the manuscript is available on our site with no access controls, subscription charges or pay-per-view fees (http://bmjopen.bmj.com).

If you have any questions on BMJ Open's open peer review process please email editorial.bmjopen@bmj.com

BMJ Open

Safety and cost analysis of selective histopathological examination following appendicectomy and cholecystectomy (FANCY study); protocol and statistical analysis plan of a prospective observational multicentre study.

Journal:	*BMJ Open*
Manuscript ID	bmjopen-2019-035912
Article Type:	Protocol
Date Submitted by the Author:	21-Nov-2019

Complete List of Authors:	Bastiaenen, Vivian; Amsterdam UMC - Locatie AMC, Department of Surgery<br>Corten, Bartholomeus; Maxima Medical Centre Location Veldhoven, Department of Surgery<br>de Savornin Lohman, Elise; Radboudumc, Department of Surgery<br>de Jonge, Joske; Tergooi Hospitals, Department of Surgery<br>Kraima, Anne; Amsterdam UMC - Locatie AMC, Department of Surgery<br>Swank, Hilko; Amsterdam UMC - Locatie AMC, Department of Surgery<br>van Vliet, Jaap; Medical Centre Haaglanden, Department of Surgery<br>van Acker, Gijs; Medical Centre Haaglanden, Department of Surgery<br>van Geloven, Anna; Tergooi Hospitals, Department of Surgery<br>in 't Hof, Klaas; Flevo Hospital, Department of Surgery<br>Koens, Lianne; Amsterdam UMC - Locatie AMC, Department of Pathology<br>de Reuver, Philip ; Radboudumc, Department of Surgery<br>van Rossem, Charles; Maasstad Hospital, Department of Surgery<br>Slooter, Gerrit; Maxima Medical Centre Location Veldhoven, Department of Surgery<br>Tanis, Pieter; Amsterdam UMC - Locatie AMC, Department of Surgery<br>Terpstra, Valeska; Medical Centre Haaglanden, Department of Pathology<br>Dijkgraaf, Marcel; Amsterdam UMC - Locatie AMC, Department of Clinical Epidemiology, Biostatistics and Bioinformatics<br>Bemelman, Willem; Amsterdam UMC - Locatie AMC, Department of Surgery

Keywords:	SURGERY, HISTOPATHOLOGY, HEALTH ECONOMICS

SCHOLARONE™
Manuscripts

TITLE PAGE

Safety and cost analysis of selective histopathological examination following appendicectomy and cholecystectomy (FANCY study); protocol and statistical analysis plan of a prospective observational multicentre study.

Vivian P. Bastiaenen[1], MD, Bartholomeus J.G.A. Corten[2], MD, Elise A.J. de Savornin Lohman[3], MD, Joske de Jonge[4], MD, Anne C. Kraima[1], MD, PhD, Hilko A. Swank[1], MD, PhD, Jaap L.P. van Vliet[5], MD, Gijs J.D. van Acker[5], MD, PhD, Anna A.W. van Geloven[4], MD, PhD, Klaas H. in 't Hof[6], MD, PhD, L. Koens[7], MD, PhD, Philip R. de Reuver[3], MD, PhD, Charles C. van Rossem[8], MD, PhD, Gerrit D. Slooter[2], MD, PhD, Pieter J. Tanis[1], MD, PhD, Valeska Terpstra[9], MD, PhD, Marcel G.W. Dijkgraaf[10], MD, PhD, Willem A. Bemelman[1], MD, PhD.

1. Department of Surgery, Amsterdam UMC, University of Amsterdam, Amsterdam, the Netherlands
2. Department of Surgery, Máxima Medical Centre, Veldhoven, the Netherlands
3. Department of Surgery, Radboud University Medical Centre, Nijmegen, the Netherlands
4. Department of Surgery, Tergooi Hospital, Hilversum, the Netherlands
5. Department of Surgery, Haaglanden Medical Centre, The Hague, the Netherlands
6. Department of Surgery, Flevo Hospital, Almere, the Netherlands
7. Department of Pathology, Amsterdam UMC, University of Amsterdam, the Netherlands
8. Department of Surgery, Maasstad Hospital, Rotterdam, the Netherlands
9. Department of Pathology, Haaglanden Medical Centre, The Hague, the Netherlands
10. Department of Clinical Epidemiology, Biostatistics and Bioinformatics, Amsterdam UMC, University of Amsterdam, Amsterdam, the Netherlands

Email addresses: v.p.bastiaenen@amsterdamumc.nl; bart.corten@mmc.nl; elise.desavorninlohman@radboudumc.nl; jdejonge@tergooi.nl; a.c.kraima@amsterdamumc.nl; h.a.swank@amsterdamumc.nl; j.van.vliet@haaglandenmc.nl; g.van.acker@haaglandenmc.nl; avangeloven@tergooi.nl; kithof@flevoziekenhuis.nl; l.koens@amsterdamumc.nl; philip.dereuver@radboudumc.nl; rossemc@maasstadziekenhuis.nl; g.slooter@mmc.nl; p.j.tanis@amsterdamumc.nl, v.terpstra@haaglandenmc.nl, m.g.dijkgraaf@amsterdamumc.nl, w.a.bemelman@amsterdamumc.nl

Corresponding author after publication (in case manuscript will be accepted):
Willem A Bemelman, MD PhD, surgeon
Department of Surgery, Amsterdam UMC, University of Amsterdam, the Netherlands
PO Box 22660, 1105 AZ Amsterdam, the Netherlands
E: W.A.Bemelman@amsterdamumc.nl, T: +31 20 732 68 18

Corresponding author during review process:
Vivian P Bastiaenen, MD, PhD Candidate
Department of Surgery, Amsterdam UMC, University of Amsterdam, the Netherlands
PO Box 22660, 1105 AZ Amsterdam, the Netherlands
E: V.P.Bastiaenen@amsterdamumc.nl, T: +31 20 566 51 99

Word count (excl. title page, abstract, tables, acknowledgements, and references): 4.702

For peer review only

ABSTRACT

Introduction

Routine histopathological examination following appendicectomy and cholecystectomy has significant financial implications and comprises a substantial portion of the pathologists' workload, while the incidence of unexpected pathology is low. The aim of the FANCY study is to investigate the oncological safety and potential cost savings of selective histopathological examination based on macroscopic assessment performed by the surgeon.

Methods and analysis

This is a Dutch multicentre prospective observational study, in which removed appendices and gallbladders will be systematically assessed by the operating surgeon for macroscopic abnormalities suspicious for malignant neoplasms. After visual inspection and digital palpation of the removed specimen, the operating surgeon will report whether macroscopic abnormalities suspicious for a malignant neoplasm are present, and if he or she believes additional microscopic examination by the pathologist is indicated. Regardless of the surgeon's assessment, all specimens will be sent for histopathological examination. In this way, routine histopathological examination can be compared to a hypothetical situation in which specimens are routinely examined by surgeons and only sent to the pathologist on indication. The two main outcomes are oncological safety and potential cost savings of a selective policy. Oncological safety of selective histopathological examination will be assessed by calculating the number of patients in whom a histopathological diagnosis of an appendiceal neoplasm or gallbladder cancer with clinical consequences benefitting the patient would have been missed. A cost analysis will be performed to quantify the potential cost savings.

Ethics and dissemination

The study protocol was reviewed by the Institutional Review Board of the Amsterdam UMC, location AMC, which decided that the Dutch Medical Research Involving Human Subjects Act (WMO) is not applicable. The study results will be disseminated through peer-reviewed publications and conference presentations. Guidelines will be revised according to the findings of the study.

Trial registration number

NCT03510923.

Keywords: Surgery, Histopathology, Health economics

For peer review only

ARTICLE SUMMARY

Strengths and limitations of this study

- This will be the first multicentre study that prospectively investigates both the oncological safety and potential cost savings of selective histopathological examination following appendicectomy and cholecystectomy in a large cohort of patients.
- The robust and transparent instruction on how to perform a proper macroscopic assessment of the removed specimens results in a uniform performed examination.
- As a result of the high participation rate of Dutch hospitals, a large number of surgeons and residents will become experienced in performing macroscopic examination of appendices and gallbladders, which will ease successful implementation of a selective policy, if oncological safety is proven.
- Patients' safety will not be compromised, since all specimens will be routinely sent for additional microscopic examination by the pathologist, regardless of the surgeon's assessment.
- Due to limited and inconclusive evidence on the prognostic impact of revisional surgery in patients with gallbladder cancer, all additional diagnostic or therapeutic procedures performed ≥T1b gallbladder were considered beneficial.

For peer review only

INTRODUCTION

In the Netherlands, approximately 16,000 appendicectomies and 22,500 cholecystectomies are performed annually. Traditionally, all removed appendices and gallbladders are microscopically examined by a pathologist to exclude the presence of a malignancy. However, most specimens show typical histopathological findings and unexpected neoplasms are only diagnosed in less than 1%.(1, 2) At the same time, histopathological examination (HPE) of the appendix and gallbladder constitutes a significant financial burden on health care, and comprises a considerable portion of the pathologists' workload. Consequently, it is debatable whether routine HPE following appendicectomy and cholecystectomy is necessary.

In the past decade, several studies suggested that a selective HPE policy might be justified.(3-8) Selective HPE entails that surgeons perform a macroscopic assessment of the removed specimen, and select specimens that require additional microscopic examination by the pathologist. Opponents fear that surgeons might not recognise neoplasms, resulting in missed diagnoses with potential disadvantageous consequences for the patient. However, it is hypothesised that tumours not detected during visual inspection or palpation are of early stage. These missed tumours are likely to be clinically inconsequential after the appendix or gallbladder have been completely resected. Most appendiceal neoplasms are neuro-endocrine tumours, which only require additional treatment if the diameter exceeds 2 cm or exhibit unfavourable histopathological characteristics.(9) In case of gallbladder cancer (GBC), an extended cholecystectomy is only indicated for stage T1b and above.(10) Unfortunately, prospective studies regarding macroscopic assessment of appendiceal specimens by the surgeon are lacking, and the few prospective studies investigating the ability of surgeons to identify macroscopic abnormalities in gallbladder specimens are limited by the small numbers of patients.(11-13)

Currently, it is still standard practice in the Netherlands to send all appendices for HPE.(14) The Dutch guideline for gallstone disease states that macroscopic normal appearing gallbladders can be refrained from HPE.(15) However, implementation of this recommendation appeared to be suboptimal, and the need for more evidence was expressed.(16) In order to draw definitive conclusions regarding safety and potential cost savings of a selective policy, a large prospective cohort of patients is required. The FANCY study will prospectively investigate the ability of surgeons to macroscopically recognise neoplasms with clinical consequences in a multicentre setting, resulting in definitive recommendations regarding the appropriate HPE policy of appendices and gallbladders.

For peer review only

METHODS AND ANALYSIS

Objectives

The primary aim of this study is to determine whether a selective HPE policy following appendicectomy and cholecystectomy for presumed benign diseases is oncologically safe. In addition, we will quantify the potential cost savings of selective HPE.

Study design

This is a Dutch multicentre prospective observational study, registered with ClinicalTrials.gov on the 27th of April 2018 (trial identification number NCT03510923). The study was designed in accordance with the principles of collaborative resident-led Snapshot research. This type of research is primarily led and conducted by surgical residents, supervised by consultants. Snapshot research is particularly suited to investigate a common condition or treatment. By generating a population-based overview, this design rapidly provides insight in current clinical practice. Previous Snapshot studies performed by the Dutch Snapshot Research Group on appendicitis, rectal cancer, and acute left-sided obstructive colon cancer demonstrated that many data can be collected in a short period of time.(17-19)

In the FANCY study, appendices and gallbladders will be systematically assessed by the operating surgeon (or surgical resident) for macroscopic abnormalities suspicious for malignant neoplasms. After visual inspection and digital palpation of the removed specimen, the operating surgeon will report on a predefined scoring form whether macroscopic abnormalities suspicious for a malignant neoplasm are present, and if he or she believes additional microscopic examination by the pathologist is indicated (Supplementary material 1). In case of suspicious macroscopic abnormalities, the surgeon is asked to describe these on the same form. Similarly, the surgeon is requested to specify the indication for HPE, if present. Regardless of the surgeon's assessment, all specimens will be sent for HPE. In this way, routine HPE can be compared to a hypothetical situation of selective HPE.

The FANCY study will be performed in sixty out of seventy-four Dutch hospitals, including academic (n=6), teaching (n=37), and non-teaching hospitals (n=17). Centres that have already implemented a selective HPE policy for gallbladder specimens may choose to either only participate in the appendix part of the FANCY study or to return to the routine policy for the duration of the study period. Hospitals will be allowed to start patient accrual after local approval has been obtained, and a site initiation visit has taken place, including a presentation of the study protocol and instructions for the macroscopic assessment of the specimens. Due to variation in completion of hospitals' local approval procedures, it was decided to open study sites in phases. Every first day of a new month, a group of hospitals will start patient accrual. To avoid bias, all hospitals will include patients for a duration of nine months, even if this means that the required sample size is exceeded.

Study population

Patients of all ages scheduled to undergo appendicectomy for appendicitis or cholecystectomy for cholecystitis or gallstone disease in the elective or non-elective setting will be included.

For peer review only

A potential subject who meets any of the following criteria will be excluded:

Appendix

- Primary indication for surgery: strong clinical/radiological suspicion or histopathological proof of an appendiceal neoplasm;
- Appendices removed as part of more extensive surgery, so-called incidental appendicectomies (e.g. right colectomy);
- Patients included in the ACCURE trial.(20)

Gallbladder

- Primary indication for surgery: strong clinical/radiological suspicion or histopathological proof of GBC;
- Gallbladder removed as part of more extensive surgery, so-called incidental cholecystectomies (e.g. Whipple procedure);
- The presence of a polyp of >10 mm on preoperative imaging.

Study observations

Macroscopic assessment

During the site initiation visits, all steps of a systematically performed macroscopic assessment will be discussed. Instruction videos showing how to perform the macroscopic assessment will be available on the study website during the entire study period.

Surgeon's macroscopic assessment of the appendix

The examination starts with inspection of the appendix and mesoappendix, followed by digital palpation. In consultation with the pathologist, it was decided that opening the appendix is prohibited, since this might impede proper HPE.

Surgeon's macroscopic assessment of the gallbladder

First, the outer surface of the gallbladder is inspected. Then, the gallbladder is incised along its longitudinal axis on the peritoneal side, leaving the cystic duct intact. After removal of stones and bile, the gallbladder mucosa is inspected and palpated. Examples of macroscopic abnormalities are masses, polyps, ulcers, cysts, hardening, irregularity and wall thickening.

HPE

All specimens will be sent to the department of pathology for macro- and microscopic assessment. HPE will be conducted according to the local protocol of the pathology department where the specimen is assessed. In general, this includes macroscopic assessment of the complete specimen, followed by microscopic assessment of samples taken from macroscopic abnormalities and the top (in case of appendices) or cystic duct margin (in case of gallbladders). If no macroscopic abnormalities are present, random samples are taken for microscopic assessment. The latest histological tumour–node–metastasis classification (TNM) of the American Joint Committee on Cancer (AJCC) applicable at the time HPE was performed was used for staging malignancies.(21)

For peer review only

Additional treatment

In case of a histopathologically proven appendiceal neoplasm or GBC, the postoperative management will be discussed in a local multidisciplinary team meeting. If it is decided that an additional resection is required, the specimens of the re-resection will be evaluated for the presence of residual tumour and positive lymph nodes, according to the local protocol.

Outcomes

All outcomes will be analysed separately for appendiceal and gallbladder specimens.

Primary outcomes

Oncological safety

Oncological safety of selective HPE will be assessed by calculating the number of patients per 1,000 examined appendices or gallbladders with the histopathological diagnosis of an appendiceal neoplasm or GBC with clinical consequences benefitting the patient that would have been missed. In case of an appendiceal neoplasm, the following consequences will be considered beneficial: 1) HPE of the re-resection specimen shows residual tumour and/or positive lymph nodes, 2) treatment with (adjuvant) systemic or local chemotherapy, radiotherapy, immunotherapy, or stem cell transplantation, and 3) palliative treatment for metastases detected during staging procedures. If an additional resection is performed following the diagnosis of an appendiceal neoplasm, and no residual tumour and/or positive lymph nodes are found during HPE, this is considered harmful due to the potential risks of surgery the patient is exposed to. For GBC, evidence on the prognostic impact of revisional surgery and adjuvant therapies is limited and inconclusive.(22) For pragmatic reasons, it was decided that all cases of GBC requiring additional diagnostic or therapeutic procedures (i.e. ≥ T1b GBC) will be considered beneficial.

To determine the number of missed diagnoses, only the specimens that would not have been sent for HPE in case of a selective policy (i.e. specimens without an indication for HPE according to the surgeon), will be analysed. In general, it is difficult to determine what incidence of missed neoplasms is acceptable to omit routine HPE. The cut off value for safety of selective HPE was chosen based on data from the Dutch national screening program for colorectal cancer (CRC). The incidence of CRC in asymptomatic patients is 0.8%, and the sensitivity of the immunochemical fecal occult blood test (iFOBT) ranges from 65% to 80%, depending on the number of screenings.(23) As a result, the diagnosis of CRC is missed in 1.6 to 2.8 per 1,000 patients. Since selective HPE implies cost savings, a reduced workload for pathologists, and less risk of overtreatment, a higher incidence of missed diagnoses is acceptable. Therefore, it was decided that selective HPE of appendices and gallbladders will be considered oncologically safe if the number of patients with a neoplasm with clinical consequences benefitting the patient that would have been missed is below 3 per 1,000 examined specimens (approximately twice the incidence of missed CRC in the screening program).

Cost analysis

The economic evaluation will be performed as a cost-minimisation analysis. In addition, a budget impact analysis of selective HPE will be performed from governmental, insurer, and hospital provider perspectives.

For peer review only

Secondary outcomes

1. The incidence of different histopathological diagnoses following appendicectomy and cholecystectomy;
2. Value of the intraoperative assessment (i.e. inspection and palpation) performed by the surgeon for detection of appendiceal neoplasms or GBC;
   a. Incidence of specimens with a recognised appendiceal neoplasm or GBC;
   b. Incidence of specimens with an unrecognised appendiceal neoplasm or GBC;
3. Indication for additional diagnostic or therapeutic procedures following histopathological diagnosis of appendiceal neoplasms or GBC and its clinical consequences, both in terms of benefit and harm;
   a. Incidence of appendiceal neoplasms and GBC requiring additional diagnostic or therapeutic procedures;
   b. Incidence of residual tumour and/or positive lymph nodes found in the re-resection specimen;
   c. Incidence of postoperative complications within 90 days after additional resection.
4. Value of the intraoperative assessment (i.e. inspection and palpation) performed by the surgeon for detection of aberrant findings other than appendiceal neoplasms and GBC.

   Appendiceal specimens

   - Incidence of parasite infection, endometriosis, granulomatosis, and other aberrant findings that would and would not have been sent for HPE.

   Gallbladder specimens

   - Incidence of adenoma, biliary intra-epithelial neoplasm, cholesterol polyp, inflammatory/hyperplastic polyp, adenomyomatosis, and other aberrant findings that would and would not have been sent for HPE.

Group size calculation

Group size calculation is based on the number of appendices and gallbladders with a tumour with clinical consequences benefitting the patient that would have been missed in case of a selective policy. According to systematic reviews, the incidences of appendiceal neoplasms and GBC are 7 per 1,000 and 4 per 1,000 patients, respectively.(1, 2) Data regarding the ability of surgeons to recognise these abnormalities and the consequences of these neoplasms is insufficient. It is however estimated that less than 1 out of 1,000 examined specimens will contain an appendiceal neoplasm or GBC with clinical consequences benefitting the patient that is not recognised by the surgeon during the macroscopic assessment. Selective HPE will be considered safe if this number does not increase to 3 per 1,000 patients. To demonstrate non-inferiority of selective compared to routine HPE, a sample size of 4,462 per cohort achieves a 84% power to detect a difference of 0.002 using a one-sided binomial test at a target significance level of 0.025, assuming a baseline and actual proportion of 0.001, and a non-inferiority limit of 0.00299. The actual significance level achieved by the Fisher's exact test is 0.021. These two cohorts (one for appendices, one for gallbladders) only include the specimens that would not have been sent for HPE in case of a selective policy. If the rate of HPE can be reduced to 20%, 5,578 patients per cohort (4,462 / 0.8) should initially be included.

For peer review only

Study organisation

The FANCY study is coordinated by a PhD candidate (VPB) under supervision of the principle investigator (WAB). The steering committee consists of seven surgeons, of whom two working in academic hospitals (PRR, PJT), and five in teaching hospitals (GJDA, AAWG, KHH, CCR, GDS), a pathologist working in an academic hospital (LK), and a pathologist working in a teaching hospital (VT), three surgical residents (ACK, HAS, JLPV), three PhD candidates (BJGAC, EAJS, JJ), and a clinical methodologist and health economist (MGWD), besides the coordinating PhD candidate and principle investigator. All local principal investigators and the residents, physician assistants and research nurses who are responsible for data collection, will be mentioned in alphabetical order as collaborators on all publications deriving from the FANCY study databases.

Data collection

The local study team of each participating hospital will be responsible for entering the prospectively collected data into an electronic case record form (CRF) build with Castor EDC, which is ISO 27001 and NEN 7510 certified.(24) Pre- and intraoperative data will be processed after surgery, and complemented with the postoperative histopathological outcomes when the pathology report is available (± two weeks after surgery). Pre- and postoperative data will be obtained from the electronic patient database (EPD) and pathology reports. Intraoperative data will be obtained from the scoring forms that will be filled in by the surgeon after examination of the specimen. In case a neoplasm is found during HPE, additional data about postoperative management, including details of additional diagnostic tests and/or treatment, postoperative morbidity and HPE of re-resection specimens, will be collected.

Monitoring of the primary endpoint

The reliability and quality of the primary endpoint will be assured in three ways: 1) revision of all pathology reports, 2) source data verification by remote monitoring of all cases with a histopathological diagnosis of appendiceal neoplasm or GBC, and 3) estimation of the incidence of appendiceal neoplasms and GBC in the group of eligible patients that were unintentionally not included.

Revision of the pathology reports

Under supervision of the two pathologists of the steering committee (LK, VT), the coordinating investigator (VPB) will revise all pathology reports. All histopathological diagnoses will be assigned to one of the predefined categories, as shown in Table 1.

Table 1. Histopathological diagnoses after appendicectomy and cholecystectomy.

APPENDICES	GALLBLADDERS
Normal appendix	Normal gallbladder
Acute inflammation	Acute inflammation
Chronic inflammation and reactive changes	Chronic inflammation and reactive changes

For peer review only

**Appendiceal neoplasms**	**Gallbladder neoplasms**
Neuro-endocrine neoplasm	Adenoma
Non-invasive epithelial neoplasm	Biliary intraepithelial neoplasm
Invasive epithelial neoplasm	Carcinoma
Lymphoma	Other malignant neoplasms
**Non-neoplastic aberrant findings**	**Non-neoplastic aberrant findings**
Parasitic infection	Cholesterol polyp
Endometriosis	Inflammatory / hyperplastic polyp
Granulomatous disease	Adenomyomatosis
Other	Other

Source data verification

Independent remote monitoring will be performed by a qualified monitor of the Clinical Research Unit of the Amsterdam UMC. Monitoring will be limited to all cases with a histopathological diagnosis of an appendiceal neoplasm or GBC. The quality assessment will focus on comparing entered data with source documents. Since no informed consent is obtained in this study, anonymised source documents of relevant patients will be supplied by the local study teams.

Estimation of the incidence of neoplasms in unintentionally not included patients

Since the macroscopic assessment of appendices and gallbladders is currently not routine practice, surgeons and residents might unintentionally forget to assess the specimen and fill in the scoring form. It is expected that the macroscopic assessment will not be performed in approximately 5-10% of all eligible patients. In order to determine whether our patient cohort is representative for all patients undergoing an appendicectomy or cholecystectomy, the incidence of appendiceal neoplasms and GBC in the group of patients that were not included has to be determined. This will be done in collaboration with PALGA, the Dutch nationwide network and registry of histopathology and cytopathology that contains pathology reports of all pathology laboratories in the Netherlands with complete coverage of reports since 1991.(25) The PALGA database will be used to assess the number of patients in the participating centres that were unintentionally not included in the FANCY study. By means of comparing the total number of appendiceal neoplasms and GBC found in the PALGA database to the study database, we will be able to identify the number of patients that were not registered in the FANCY study. In collaboration with a staff member of PALGA, the individual pathology reports (without patient identifying information) of these patients will be checked for exclusion criteria. Consequently, the (estimated) incidence of appendiceal neoplasms and GBC in the group of unintentionally not included patients will be known.

Cleaning and locking of the database

The database will be locked and exported for statistical analysis as soon as all data is entered, and all missing items are checked with the local study team. After locking, the database will be archived in a licensed repository.

For peer review only

Predefined statistical analysis plan

General principles

The analyses will be performed after data entry is completed, monitoring and cleaning of the data has been performed, and the statistical analysis plan is accepted for publication. For the primary analyses, all patients who underwent an appendicectomy for appendicitis or cholecystectomy for cholecystitis or gallstone disease will be included. All analyses described below will be performed using the latest version of SPSS statistics (IBM Corp., Armonk, NY, USA) at the time of analysis.

Baseline characteristics

Baseline characteristics will be expressed as medians and interquartile ranges (IQR), or counts and percentages. Baseline characteristics will be presented as shown in Table 2 (appendices) and Table 3 (gallbladders).

Table 2. Baseline characteristics (appendices).

		**Total (n=)**
Age, years		Median (IQR)
Sex, n (%)	Female	n (% of 'Total')
	Male	n (% of 'Total')
Preoperative imaging, n (%)	Ultrasound	n (% of 'Total')
	Ultrasound + CT	n (% of 'Total')
	Ultrasound + MRI	n (% of 'Total')
	Ultrasound + CT + MRI	n (% of 'Total')
	CT	n (% of 'Total')
	MRI	n (% of 'Total')
	CT + MRI	n (% of 'Total')
	No preoperative imaging	n (% of 'Total')
Hospital	Academic hospital	n (% of 'Total')
	Teaching hospital	n (% of 'Total')
	Non-teaching hospital	n (% of 'Total')
Macroscopic assessment performed by	Surgeon	n (% of 'Total')
	Resident	n (% of 'Total')
	Both	n (% of 'Total')

CT, computed tomography; IQR, interquartile range; MRI, magnetic resonance imaging

For peer review only

Table 3. Baseline characteristics (gallbladder).

		Total (n=)
Age, years		Median (IQR)
Sex, n (%)	Female	n (% of 'Total')
	Male	n (% of 'Total')
Preoperative diagnosis, n (%)	Cholecystitis	n (% of 'Total')
	Symptomatic cholelithiasis	n (% of 'Total')
Preoperative imaging, n (%)	Ultrasound	n (% of 'Total')
	Ultrasound + CT	n (% of 'Total')
	Ultrasound + MRI	n (% of 'Total')
	Ultrasound + CT + MRI	n (% of 'Total')
	CT	n (% of 'Total')
	MRI	n (% of 'Total')
	CT + MRI	n (% of 'Total')
	Other	n (% of 'Total')
	No preoperative imaging	n (% of 'Total')
Surgical setting	Acute	n (% of 'Total')
	Elective	n (% of 'Total')
Hospital	Academic hospital	n (% of 'Total')
	Teaching hospital	n (% of 'Total')
	Non-teaching hospital	n (% of 'Total')
Macroscopic assessment performed by	Surgeon	n (% of 'Total')
	Resident	n (% of 'Total')
	Both	n (% of 'Total')

CT, computed tomography; IQR, interquartile range; MRI, magnetic resonance imaging

Primary outcomes

Oncological safety

The number of patients with a histopathological diagnosis of an appendiceal neoplasm or GBC with clinical consequences benefitting the patient will be reported in absolute numbers and percentages, and as number per 1,000 examined specimens for both strategies of HPE (routine and selective). The data will be presented as in Figure 1 (appendices) and Figure 2 (gallbladders). For analysis of the primary outcome, only the specimens that would not have been sent for HPE in case of a selective policy (i.e. specimens without an indication for HPE according to the surgeon), will be analysed. A selective policy will be considered safe, if, following an exact test, the one-sided upper limit at a 97.5% confidence level of the proportion of missed malignancies falls below 3 per 1,000 examined specimens. The influence of the assessor of the specimen (surgeon vs. resident) and hospital (academic hospital vs. teaching hospital vs. non-teaching hospital) on the primary outcome will be assessed with Poisson regression.

Costs

1) Economic evaluation

Considering that – under the non-inferiority hypothesis – 1 or 2 per 1,000 patients at maximum will experience health consequences from routine HPE, and 3 to 4 per 1,000 patients may experience transient harm following unnecessary additional treatment, differences in effectiveness between the routine and selective strategy can best

For peer review only

be addressed qualitatively (e.g. by case reports). Quantitatively, a cost analysis from a health care provider will be the main focus of research.

During the study, all specimens will be sent for HPE, so the selective policy will not be observed. The comparison between both strategies will therefore be done through decision tree analysis. Observed distributions of histopathological findings under the routine policy will be used to define chance nodes in the reference tree of the model. Alternatively, both the distribution of histopathological findings in specimens that would have been sent for HPE as the distribution of histopathological findings in specimen that would not have been sent for HPE in case of a selective policy will be used to define chance nodes in the other main model tree.

In addition to the initial HPE, costs of other resources (e.g. additional treatment, additional HPE, hospital stay) will be assigned to each end node in the model. If costs were observed under routine examination, but the specimen would not have been sent for HPE in case of a selective policy, they will be ignored if related to unnecessary use of health care resources. However, if these costs were justified, and the specimen would not have been sent for HPE in case of a selective policy, it will be assumed that these costs would nevertheless be generated at a later stage during the disease course, and thus included in the decision tree. In addition, a scenario analysis will be run with a 50% surplus penalty of these costs to compensate for yet unobserved extra costs of delayed health care at a later disease stage. Unit costing of hospital resources will be based on the Erasmus University Rotterdam / National Health Care Institute guideline for costing in health care research.(26) If specific unit costs are lacking in the guideline, local bottom-up or top-down costing initiatives in participating hospitals (e.g. Amsterdam UMC) will be used.

All probabilities at the chance nodes will be assumed Beta-distributed. Multiple theoretical distributions will be assessed for fitting the (observed) distributions of health care costs at the end nodes of the decision tree. If theoretical fits seem insufficient, a uniform distribution will be defined for the (observed) cost data. Monte Carlo simulation will be applied based on 25,000 draws from each distribution of input parameters. It is expected that a time horizon for the cost analysis of six months is sufficient to reliably estimate the cost difference between routine and selective HPE. Separate models will be built for the analyses of appendices and gallbladders.

2) Budget Impact Analysis

The budget impact analysis of selective HPE will be performed from governmental, insurance, and hospital provider perspectives for a four-year budget period, starting with the first full budget year after completion of the trial. The budget impact will be expressed in millions of euros. Primarily, the budget for care by medical specialists (code 0303), such as pathologists and surgeons, will be affected. For all perspectives, the reimbursement guidelines from the Dutch Healthcare Authority will be applied to the estimate actual expenses.

In case of negotiable reimbursement levels, the 10% trimmed mean purchase price per unit as provided by www.opendisdata.nl (e.g. DBC-code 119599010) will be used. Data on the incidences of performed appendicectomies and cholecystectomies will be gathered from public data sources (www.opendisdata.nl;

For peer review only

www.statline.cbs.nl), and linearly extrapolated to forecast the numbers during the period for budget impact analysis.

Secondary outcomes

All incidences of histopathological diagnoses will be reported in absolute numbers and percentages. The incidence of different histopathological diagnoses will be presented as shown in Table 4 (appendices) and Table 5 (gallbladders). These tables will also provide information on which specimens would and would not have been sent for HPE in case of a selective policy. Table 6 will show whether or not specimens containing an appendiceal neoplasm or GBC were reported as suspicious by the surgeon and whether or not the surgeon believed HPE was indicated. Details on all patients with an appendiceal neoplasm or GBC will be presented as proposed in Table 7 (appendices) and Table 8 (gallbladders).

Table 4. Histopathological diagnoses after appendicectomy for appendicitis.

			**Total (n=)**	**Indication for HPE[a] (n=)**	**No indication for HPE[a] (n=)**
Histopathological diagnosis		Normal appendix	n (% of 'Total)	n	n
		Acute inflammation	n (% of 'Total')	n[b]	n[c]
		Chronic inflammation and reactive changes	n (% of 'Total')	n	n
	Appendiceal neoplasms	Neuro-endocrine neoplasm	n (% of 'Total')	n	n
		Non-invasive epithelial neoplasm	n (% of 'Total')	n	n
		Invasive epithelial neoplasm	n (% of 'Total')	n	n
		Lymphoma	n (% of 'Total')	n	n
	Non-neoplastic aberrant findings	Parasitic infection	n (% of 'Total')	n	n
		Endometriosis	n (% of 'Total')	n	n
		Granulomatous disease	n (% of 'Total')	n	n
		Other	n (% of 'Total')	n	n

HPE, histopathological examination

a. According to the operating surgeon or surgical resident.
b. Uncomplicated acute appendicitis (n=), complicated acute appendicitis (n=); as reported in pathology report.
c. Uncomplicated acute appendicitis (n=), complicated acute appendicitis (n=); as reported in pathology report.

For peer review only

Table 5. Histopathological diagnoses after cholecystectomy for presumed benign gallbladder diseases.

			Total (n=)	Indication for HPE[a] (n=)	No indication for HPE[a] (n=)
Histopathological diagnosis		Normal gallbladder	n (% of 'Total)	n	n
		Acute inflammation	n (% of 'Total')	n	n
		Chronic inflammation and reactive changes	n (% of 'Total')	n	n
	Gallbladder neoplasms	Adenoma	n (% of 'Total')	n	n
		Biliary intraepithelial neoplasm	n (% of 'Total')	n	n
		Carcinoma	n (% of 'Total')	n	n
		Other malignant neoplasms	n (% of 'Total')	n[b]	n[c]
	Non-neoplastic aberrant findings	Cholesterol polyp	n (% of 'Total')	n	n
		Inflammatory / hyperplastic polyp	n (% of 'Total')	n	n
		Adenomyomatosis	n (% of 'Total')	n	n
		Other	n (% of 'Total')	n	n
HPE, histopathological examination					
a. According to the operating surgeon or surgical resident.<br>b. Details on histology.<br>c. Details on histology.					

Table 6. Value of the intraoperative assessment by the surgeon for detection of appendiceal neoplasms / GBC

	Appendiceal neoplasm / GBC (n=)	No appendiceal neoplasm / GBC (n=)	Total (n=)
**Presence of abnormalities suspicious for malignancy**	n (% of 'Appendiceal neoplasm / GBC)	n (% of 'No appendiceal neoplasm / GBC)	n (% of 'Total')
**Indication for HPE**	n (% of 'Appendiceal neoplasm / GBC)	n (% of ' No appendiceal neoplasm / GBC)	n (% of 'Total')
GBC, gallbladder cancer; HPE, histopathological examination			

For peer review only

Table 7. Details of patients with a histopathological diagnosis of an appendiceal neoplasm.

Case	Sex, Age	Preoperative imaging	Assessor	Macroscopic abnormalities suspicious for neoplasm	Indication for HPE according to surgeon	Histopathological diagnosis	Additional diagnostic and/or therapeutic procedures	Remaining tumour tissue	Positive lymph nodes	90-day complications
1										
2										
3										
…										

Table 8. Details of patients with a histopathological diagnosis of GBC.

Case	Sex, Age	Preoperative imaging	Preoperative diagnosis	Surgical setting	Assessor	Macroscopic abnormalities suspicious for neoplasm	Indication for HPE according to surgeon	Histopathological diagnosis	Additional diagnostic and/or therapeutic procedures	Residual disease	90-day complications
1											
2											
3											
…											

Several exploratory subgroup analyses will be performed. For appendiceal specimens, the influence of age (adults vs. children) on the incidence of different histopathological diagnoses will be evaluated and reported in a similar way as shown in Table 4. For gallbladder specimens, a subgroup analysis on the influence of preoperative diagnosis (cholecystitis vs. gallstone disease) on the incidence of different histopathological diagnoses will be performed. Furthermore, the influence of the assessor (surgeon vs. resident) and hospital (academic hospital vs. teaching hospital vs. non-teaching hospital) on the rate of specimens that would have been sent for HPE will be reported.

Current status of the study

The study was registered with ClinicalTrials.gov on the 27th of April 2018 (trial identification number NCT03510923) and in the Netherlands Trial Register on the 16th of April 2018 under number NTR7151 (www.trialregister.nl). Recruitment of patients started in May 2018. At time of submission, November 2019, 53 of 60 hospitals have finished the 13 months period of data collection (nine months accrual followed by four months for data entry) and 6,902 and 8,387 patients have been included in the appendices and gallbladders databases, respectively.

Manuscripts and authorship

The steering committee of the FANCY study will share the results irrespective of the outcomes. The outcomes as described in this protocol will be reported in two manuscripts, one for the appendices and one for the gallbladders. These manuscripts will be submitted with the steering committee as co-authors and all other investigators as collaborators. The coordinating investigator (VPB) and principal investigator (WAB) will be first and senior author

For peer review only

on both manuscripts, respectively. If the results of the economic evaluation are reported separately, senior authorship for this manuscript will be shared by WAB and MGWD. For the appendices manuscript, the other PhD candidates will be second (JJ), third (BJGAC) and fourth author (EAJS). If possible, JLPV, EAJS and BJAGC will share second authorship on the gallbladder manuscript. The other members of the steering committee will be co-authors on both publications. All local principal investigators and the residents, physician assistants and research nurses who were responsible for data collection, will be mentioned in alphabetical order as collaborators. All efforts will be made to link the collaborators to the final publications in indexed databases.

Patient and public involvement

Patients and public were not involved in designing the study.

For peer review only

ETHICS AND DISSEMINATION

Ethical aspects and informed consent

This study will be performed in accordance with the principles of Good Clinical Practice, the Dutch Agreement on Medical Treatment Act (WGBO) and the European General Data Protection Regulation. The study protocol was reviewed by the Institutional Review Board of the Amsterdam UMC, location AMC, which decided that the Dutch Medical Research Involving Human Subjects Act (WMO) is not applicable. In all participating centres, approval for execution of the FANCY study will be obtained from the local Institutional Review Board before the start of inclusion of patients.

In the FANCY study, a large number of patients will be included in a relatively short period of time. After consultation with the legal department of the Amsterdam UMC, it was decided that no written informed consent will be requested for the use of patients' data. Obtaining written informed consent of all included patients during the usually short hospital admission would be futile and impede the execution of this study. Participation in the FANCY study does not have any treatment consequences for patients, as there is no change in current clinical practice. Patients will easily postpone their decision on participation. Moreover, it was suggested that certain patient groups (e.g. young patients, patients with a complicated postoperative course, patients with histopathological findings requiring additional hospital visits) tend to provide informed consent more often, which would introduce selection bias. A deferred consent procedure including a phone call in the postoperative period was considered but deemed impractical due to the large number of health care providers involved. For these reasons, it was decided that the extensive effort to obtain informed consent does not compete with the relatively small amount of non-identifiable data that is collected in the FANCY study. Alternatively, patients will be offered the opportunity to refuse the use of their data by using an opt-out procedure. All patients that underwent an appendicectomy or cholecystectomy will receive a leaflet with brief information about the study. It will be explained that all data will be extracted from the patient's charts followed by deidentification and no additional investigations are required. When a patient or its relatives object to participate, the patient will be excluded from the study and data will not be entered into the database.

Dissemination

During the study, all collaborators will be updated about the progress of the study by monthly newsletters. The results of the FANCY study will be presented at national and international conferences and submitted for publication in an international peer-reviewed scientific journal. The Dutch Surgical Society (NVVH), which is responsible for revision of the guidelines, recognises the relevance of this research and supports the implementation of the results. As secretary of the Board of Directors of the NVVH (GJDA) and chairman of the guideline committee 'Appendicitis' (CCR), two of our steering committee members are involved in the revision of the guidelines, which ensures that the guidelines will be adjusted according to the results of the FANCY study.

For peer review only

ACKNOWLEDGEMENTS

None.

AUTHOR CONTRIBUTIONS

VPB, MGWD and WAB have made substantial contributions to the conception and design of this study and have been involved in drafting the manuscript or revising it critically for important intellectual content. BJGA have made substantial contributions to the design of this study and have been involved in drafting the manuscript. EAJS, JJ, ACK, HAS, JLPV, GJDA, AAWG, KHH, LK, PRR, CCR, GDS, PJT and VT have made substantial contributions to the design of this study and the organisation of this trial and have revised the manuscript critically for important intellectual content. All authors have given final approval of the version to be published.

FUNDING

This investigator initiated study was supported by the Netherlands Organisation for Health Research and Development (ZonMw) grant number 843002822, of which the Dutch Ministry of Health, Welfare and Sports and the Dutch Organisation for Scientific Research are the main commissioning organisations. ZonMw has not played a role in designing the study and data collection, nor will it influence the analysis and writing process of the manuscripts.

COMPETING INTERESTS

None declared.

PATIENT CONSENT FOR PUBLICATION

Not required.

For peer review only

REFERENCES

1. Swank HA, Eshuis EJ, Ubbink DT, et al. Is routine histopathological examination of appendectomy specimens useful? A systematic review of the literature. *Colorectal Dis*. 2011;13(11):1214-21.
2. Swank HA, Mulder IM, Hop WC, et al. Routine histopathology for carcinoma in cholecystectomy specimens not evidence based: a systematic review. *Surg Endosc*. 2013;27(12):4439-48.
3. Khan RA, Ghani I, Chana RS. Routine histopathological examination of appendectomy specimens in children: is there any rationale? *Pediatr Surg Int*. 2011;27(12):1313-5.
4. Jahan B, Najeeb S, Shaikh AW. Acute appendicitis, correlating histopathological findings with clinical - is histopathology needed for all? *PJMHS*. 2016;10(1):118-21.
5. Van Vliet JLP, Van Gulik TM, Verbeek PCM. Is it necessary to send gallbladder specimens for routine histopathological examination after cholecystectomy? The use of macroscopic examination. *Digestive Surgery*. 2014;30(4-6):472-5.
6. Emmett CD, Barrett P, Gilliam AD, et al. Routine versus selective histological examination after cholecystectomy to exclude incidental gallbladder carcinoma. *Ann R Coll Surg Engl*. 2015;97(7):526-9.
7. Olthof PB, Metman MJH, de Krijger RR, et al. Routine Pathology and Postoperative Follow-Up are Not Cost-Effective in Cholecystectomy for Benign Gallbladder Disease. *World J Surg*. 2018;42(10):3165-70.
8. Deng YL, Xiong XZ, Zhou Y, et al. Selective histology of cholecystectomy specimens--is it justified? *J Surg Res*. 2015;193(1):196-201.
9. Pape UF, Niederle B, Costa F, et al. ENETS Consensus Guidelines for Neuroendocrine Neoplasms of the Appendix (Excluding Goblet Cell Carcinomas). *Neuroendocrinology*. 2016;103(2):144-52.
10. Aloia TA, Jarufe N, Javle M, et al. Gallbladder cancer: expert consensus statement. *HPB (Oxford)*. 2015;17(8):681-90.
11. Romero-Gonzalez RJ, Garza-Flores A, Martinez-PerezMaldonado L, et al. Gallbladder selection for histopathological analysis based on a simple method: a prospective comparative study. *Annals of the Royal College of Surgeons of England*. 2012;94(3):159-64.
12. Tayeb M, Rauf F, Ahmad K, et al. Is it necessary to submit grossly normal looking gall bladder specimens for histopathological examination? *Asian Pac J Cancer Prev*. 2015;16(4):1535-8.
13. Corten B, Alexander S, van Zwam PH, et al. Outcome of Surgical Inspection of the Gallbladder in Relation to Final Pathology. *J Gastrointest Surg*. 2019;23(6):1130-4.
14. NVVH. Evidence-based richtlijn. Nederlandse Vereniging voor Heelkunde, Acute appendicitis. 2019.
15. NVVH. Evidence-based richtlijn. Nederlandse Vereniging voor Heelkunde, Galsteenlijden. 2016.
16. Corten B, Leclercq WKG, Dejong CH, et al. Selective Histological Examination After Cholecystectomy: An Analysis of Current Daily Practice in The Netherlands. *World J Surg*. 2019.
17. van Rossem CC, Bolmers MD, Schreinemacher MH, et al. Prospective nationwide outcome audit of surgery for suspected acute appendicitis. *Br J Surg*. 2016;103(1):144-51.
18. Borstlap WAA, Westerduin E, Aukema TS, et al. Anastomotic Leakage and Chronic Presacral Sinus Formation After Low Anterior Resection: Results From a Large Cross-sectional Study. *Ann Surg*. 2017;266(5):870-7.
19. Amelung FJ, Borstlap WAA, Consten ECJ, et al. Propensity score-matched analysis of oncological outcome between stent as bridge to surgery and emergency resection in patients with malignant left-sided colonic obstruction. *Br J Surg*. 2019;106(8):1075-86.

For peer review only

20. Gardenbroek TJ, Pinkney TD, Sahami S, et al. The ACCURE-trial: the effect of appendectomy on the clinical course of ulcerative colitis, a randomised international multicenter trial (NTR2883) and the ACCURE-UK trial: a randomised external pilot trial (ISRCTN56523019). *BMC Surg*. 2015;15:30.
21. Amin MB, Edge S, Greene F, et al. AJCC Cancer Staging Manual (8th edition). 2017.
22. Soreide K, Guest RV, Harrison EM, et al. Systematic review of management of incidental gallbladder cancer after cholecystectomy. *Br J Surg*. 2019;106(1):32-45.
23. Wiersma T, de Wit N, Drenthen T. NHG-Standpunt Bevolkingsonderzoek darmkanker. 2013.
24. Castor EDC. Castor Electronic Data Capture 2019 [27 Aug. 2019]. Available from: https://castoredc.com.
25. Casparie M, Tiebosch AT, Burger G, et al. Pathology databanking and biobanking in The Netherlands, a central role for PALGA, the nationwide histopathology and cytopathology data network and archive. *Cell Oncol*. 2007;29(1):19-24.
26. Zorginstituut N. Richtlijn voor het uitvoeren van economische evaluaties in de gezondheidszorg. Diemen. 2015.

For peer review only

FIGURE LEGENDS

Figure 1. Number of patients with an appendiceal neoplasm with clinical consequences benefitting the patient that would have been diagnosed (green box) and missed (red box) in case of a selective policy. The gray dotted line indicates the total number of patients benefitting from clinical consequences of an appendiceal neoplasm, that would have been diagnosed in case of a routine policy.

Figure 2. Number of patients with gallbladder cancer with clinical consequences benefitting the patient that would have been diagnosed (green box) and missed (red box) in case of a selective policy. The gray dotted line indicates the total number of patients benefitting from clinical consequences of gallbladder cancer, that would have been diagnosed in case of a routine policy.

For peer review only

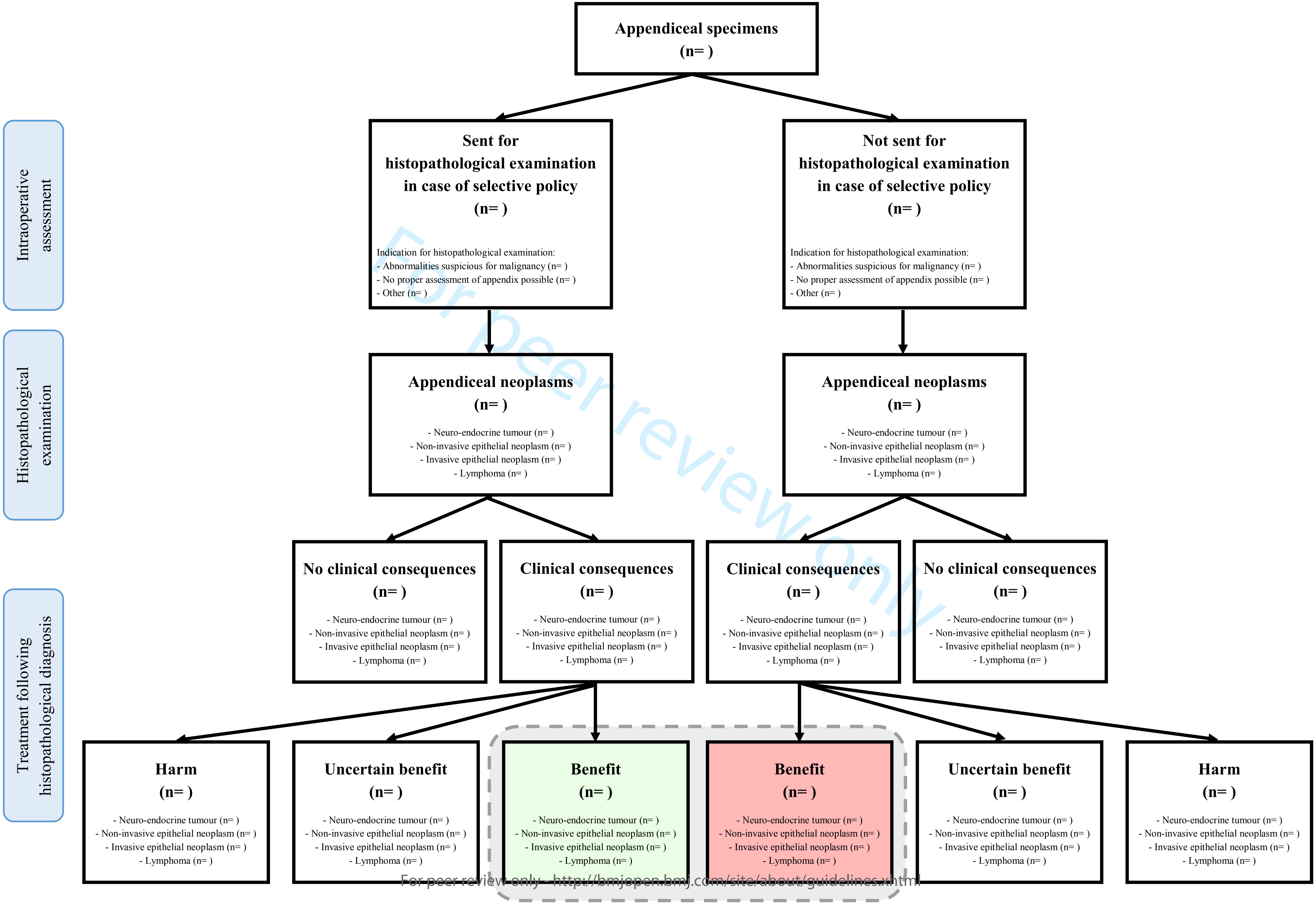

For peer review only - http://bmjopen.bmj.com/site/about/guidelines.xhtml

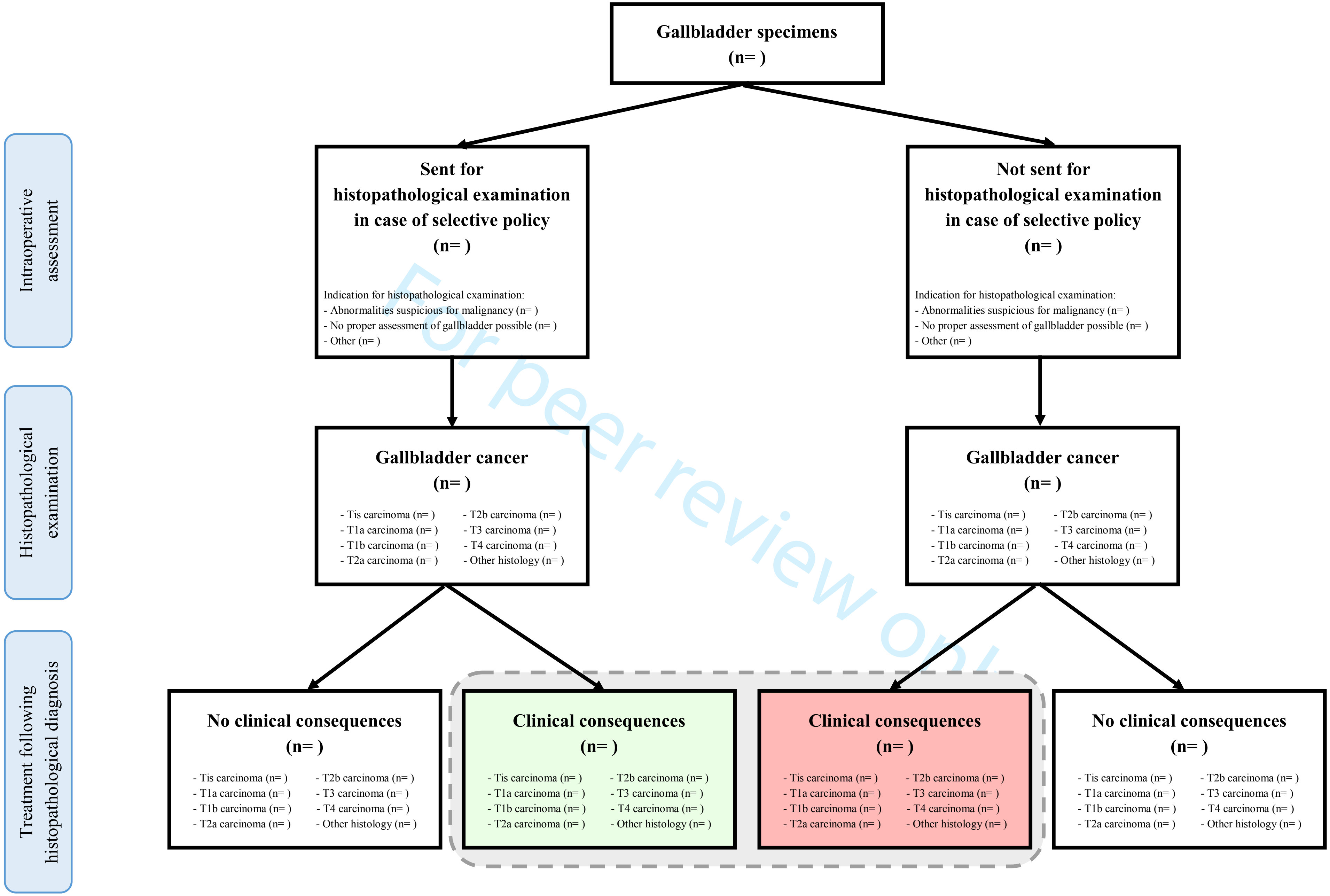

For peer review only

SUPPLEMENTARY MATERIAL 1

Questions of the scoring form 'Macroscopic examination of the appendix'

1) Who performed the macroscopic examination of the appendix?
Multiple answers possible
O Surgeon
O Surgical resident

2) Are there any macroscopic abnormalities suspicious for a malignant neoplasm during visual inspection and/or digital palpation?
O Yes (please specify below)
O No
O Proper assessment is not possible due to inflammation

2a. If yes, specify the macroscopic abnormalities
Multiple answers possible
O Visible tumour
O Palpable tumour
O Other, namely …

3) Do you believe additional histopathological examination by the pathologist is indicated?
O Yes, because of the above-mentioned abnormalities.
O Yes, because proper assessment of the appendix was not possible.
O Yes, because …
O No

Questions of the scoring form 'Macroscopic examination of the gallbladder'

1) Who performed the macroscopic examination of the gallbladder?
Multiple answers possible
O Surgeon
O Surgical resident

2) Are there any macroscopic abnormalities suspicious for a malignant neoplasm during visual inspection and/or digital palpation?
O Yes (please specify below)
O No

2a. If yes, specify the macroscopic abnormalities
Multiple answers possible
O Ulcer
O Cyst
O Hardening
O Irregularity
O Wall thickening
O Polyp
O Tumour suspicious for malignancy
O Other, namely …

3) Do you believe additional histopathological examination by the pathologist is indicated?
O Yes, because of the above-mentioned abnormalities.
O Yes, because …
O No

For peer review only

BMJ Open

Safety and cost analysis of selective histopathological examination following appendicectomy and cholecystectomy (FANCY study); protocol and statistical analysis plan of a prospective observational multicentre study.

Journal:	*BMJ Open*
Manuscript ID	bmjopen-2019-035912.R1
Article Type:	Protocol
Date Submitted by the Author:	26-Nov-2019

Complete List of Authors:	Bastiaenen, Vivian; Amsterdam UMC - Locatie AMC, Department of Surgery<br>Corten, Bartholomeus; Maxima Medical Centre Location Veldhoven, Department of Surgery<br>de Savornin Lohman, Elise; Radboudumc, Department of Surgery<br>de Jonge, Joske; Tergooi Hospitals, Department of Surgery<br>Kraima, Anne; Amsterdam UMC - Locatie AMC, Department of Surgery<br>Swank, Hilko; Amsterdam UMC - Locatie AMC, Department of Surgery<br>van Vliet, Jaap; Medical Centre Haaglanden, Department of Surgery<br>van Acker, Gijs; Medical Centre Haaglanden, Department of Surgery<br>van Geloven, Anna; Tergooi Hospitals, Department of Surgery<br>in 't Hof, Klaas; Flevo Hospital, Department of Surgery<br>Koens, Lianne; Amsterdam UMC - Locatie AMC, Department of Pathology<br>de Reuver, Philip ; Radboudumc, Department of Surgery<br>van Rossem, Charles; Maasstad Hospital, Department of Surgery<br>Slooter, Gerrit; Maxima Medical Centre Location Veldhoven, Department of Surgery<br>Tanis, Pieter; Amsterdam UMC - Locatie AMC, Department of Surgery<br>Terpstra, Valeska; Medical Centre Haaglanden, Department of Pathology<br>Dijkgraaf, Marcel; Amsterdam UMC - Locatie AMC, Department of Clinical Epidemiology, Biostatistics and Bioinformatics<br>Bemelman, Willem; Amsterdam UMC - Locatie AMC, Department of Surgery

**Primary Subject Heading**:	Surgery
Secondary Subject Heading:	Pathology
Keywords:	SURGERY, HISTOPATHOLOGY, HEALTH ECONOMICS

SCHOLARONE™
Manuscripts

TITLE PAGE

Safety and cost analysis of selective histopathological examination following appendicectomy and cholecystectomy (FANCY study); protocol and statistical analysis plan of a prospective observational multicentre study.

Vivian P. Bastiaenen[1], MD, Bartholomeus J.G.A. Corten[2], MD, Elise A.J. de Savornin Lohman[3], MD, Joske de Jonge[4], MD, Anne C. Kraima[1], MD, PhD, Hilko A. Swank[1], MD, PhD, Jaap L.P. van Vliet[5], MD, Gijs J.D. van Acker[5], MD, PhD, Anna A.W. van Geloven[4], MD, PhD, Klaas H. in 't Hof[6], MD, PhD, L. Koens[7], MD, PhD, Philip R. de Reuver[3], MD, PhD, Charles C. van Rossem[8], MD, PhD, Gerrit D. Slooter[2], MD, PhD, Pieter J. Tanis[1], MD, PhD, Valeska Terpstra[9], MD, PhD, Marcel G.W. Dijkgraaf[10], MD, PhD, Willem A. Bemelman[1], MD, PhD.

1. Department of Surgery, Amsterdam UMC, University of Amsterdam, Amsterdam, the Netherlands
2. Department of Surgery, Máxima Medical Centre, Veldhoven, the Netherlands
3. Department of Surgery, Radboud University Medical Centre, Nijmegen, the Netherlands
4. Department of Surgery, Tergooi Hospital, Hilversum, the Netherlands
5. Department of Surgery, Haaglanden Medical Centre, The Hague, the Netherlands
6. Department of Surgery, Flevo Hospital, Almere, the Netherlands
7. Department of Pathology, Amsterdam UMC, University of Amsterdam, the Netherlands
8. Department of Surgery, Maasstad Hospital, Rotterdam, the Netherlands
9. Department of Pathology, Haaglanden Medical Centre, The Hague, the Netherlands
10. Department of Clinical Epidemiology, Biostatistics and Bioinformatics, Amsterdam UMC, University of Amsterdam, Amsterdam, the Netherlands

Email addresses: v.p.bastiaenen@amsterdamumc.nl; bart.corten@mmc.nl; elise.desavorninlohman@radboudumc.nl; jdejonge@tergooi.nl; a.c.kraima@amsterdamumc.nl; h.a.swank@amsterdamumc.nl; j.van.vliet@haaglandenmc.nl; g.van.acker@haaglandenmc.nl; avangeloven@tergooi.nl; kithof@flevoziekenhuis.nl; l.koens@amsterdamumc.nl; philip.dereuver@radboudumc.nl; rossemc@maasstadziekenhuis.nl; g.slooter@mmc.nl; p.j.tanis@amsterdamumc.nl, v.terpstra@haaglandenmc.nl, m.g.dijkgraaf@amsterdamumc.nl, w.a.bemelman@amsterdamumc.nl

Corresponding author after publication (in case manuscript will be accepted):
Willem A Bemelman, MD PhD, surgeon
Department of Surgery, Amsterdam UMC, University of Amsterdam, the Netherlands
PO Box 22660, 1105 AZ Amsterdam, the Netherlands
E: W.A.Bemelman@amsterdamumc.nl, T: +31 20 732 68 18

Corresponding author during review process:
Vivian P Bastiaenen, MD, PhD Candidate
Department of Surgery, Amsterdam UMC, University of Amsterdam, the Netherlands
PO Box 22660, 1105 AZ Amsterdam, the Netherlands
E: V.P.Bastiaenen@amsterdamumc.nl, T: +31 20 566 51 99

Word count (excl. title page, abstract, tables, acknowledgements, and references): 4.702

For peer review only

ABSTRACT

Introduction

Routine histopathological examination following appendicectomy and cholecystectomy has significant financial implications and comprises a substantial portion of the pathologists' workload, while the incidence of unexpected pathology is low. The aim of the FANCY study is to investigate the oncological safety and potential cost savings of selective histopathological examination based on macroscopic assessment performed by the surgeon.

Methods and analysis

This is a Dutch multicentre prospective observational study, in which removed appendices and gallbladders will be systematically assessed by the operating surgeon for macroscopic abnormalities suspicious for malignant neoplasms. After visual inspection and digital palpation of the removed specimen, the operating surgeon will report whether macroscopic abnormalities suspicious for a malignant neoplasm are present, and if he or she believes additional microscopic examination by the pathologist is indicated. Regardless of the surgeon's assessment, all specimens will be sent for histopathological examination. In this way, routine histopathological examination can be compared to a hypothetical situation in which specimens are routinely examined by surgeons and only sent to the pathologist on indication. The two main outcomes are oncological safety and potential cost savings of a selective policy. Oncological safety of selective histopathological examination will be assessed by calculating the number of patients in whom a histopathological diagnosis of an appendiceal neoplasm or gallbladder cancer with clinical consequences benefitting the patient would have been missed. A cost analysis will be performed to quantify the potential cost savings.

Ethics and dissemination

The study protocol was reviewed by the Institutional Review Board of the Amsterdam UMC, location AMC, which decided that the Dutch Medical Research Involving Human Subjects Act (WMO) is not applicable. The study results will be disseminated through peer-reviewed publications and conference presentations. Guidelines will be revised according to the findings of the study.

Trial registration number

NCT03510923.

Keywords: Surgery, Histopathology, Health economics

For peer review only

ARTICLE SUMMARY

Strengths and limitations of this study

- This will be the first multicentre study that prospectively investigates both the oncological safety and potential cost savings of selective histopathological examination following appendicectomy and cholecystectomy in a large cohort of patients.
- The robust and transparent instruction on how to perform a proper macroscopic assessment of the removed specimens results in a uniform performed examination.
- As a result of the high participation rate of Dutch hospitals, a large number of surgeons and residents will become experienced in performing macroscopic examination of appendices and gallbladders, which will ease successful implementation of a selective policy, if oncological safety is proven.
- Patients' safety will not be compromised, since all specimens will be routinely sent for additional microscopic examination by the pathologist, regardless of the surgeon's assessment.
- Due to limited and inconclusive evidence on the prognostic impact of revisional surgery in patients with gallbladder cancer, all additional diagnostic or therapeutic procedures performed ≥T1b gallbladder were considered beneficial.

For peer review only

INTRODUCTION

In the Netherlands, approximately 16,000 appendicectomies and 22,500 cholecystectomies are performed annually. Traditionally, all removed appendices and gallbladders are microscopically examined by a pathologist to exclude the presence of a malignancy. However, most specimens show typical histopathological findings and unexpected neoplasms are only diagnosed in less than 1%.(1, 2) At the same time, histopathological examination (HPE) of the appendix and gallbladder constitutes a significant financial burden on health care, and comprises a considerable portion of the pathologists' workload. Consequently, it is debatable whether routine HPE following appendicectomy and cholecystectomy is necessary.

In the past decade, several studies suggested that a selective HPE policy might be justified.(3-8) Selective HPE entails that surgeons perform a macroscopic assessment of the removed specimen, and select specimens that require additional microscopic examination by the pathologist. Opponents fear that surgeons might not recognise neoplasms, resulting in missed diagnoses with potential disadvantageous consequences for the patient. However, it is hypothesised that tumours not detected during visual inspection or palpation are of early stage. These missed tumours are likely to be clinically inconsequential after the appendix or gallbladder have been completely resected. Most appendiceal neoplasms are neuro-endocrine tumours, which only require additional treatment if the diameter exceeds 2 cm or exhibit unfavourable histopathological characteristics.(9) In case of gallbladder cancer (GBC), an extended cholecystectomy is only indicated for stage T1b and above.(10) Unfortunately, prospective studies regarding macroscopic assessment of appendiceal specimens by the surgeon are lacking, and the few prospective studies investigating the ability of surgeons to identify macroscopic abnormalities in gallbladder specimens are limited by the small numbers of patients.(11-13)

Currently, it is still standard practice in the Netherlands to send all appendices for HPE.(14) The Dutch guideline for gallstone disease states that macroscopic normal appearing gallbladders can be refrained from HPE.(15) However, implementation of this recommendation appeared to be suboptimal, and the need for more evidence was expressed.(16) In order to draw definitive conclusions regarding safety and potential cost savings of a selective policy, a large prospective cohort of patients is required. The FANCY study will prospectively investigate the ability of surgeons to macroscopically recognise neoplasms with clinical consequences in a multicentre setting, resulting in definitive recommendations regarding the appropriate HPE policy of appendices and gallbladders.

For peer review only

METHODS AND ANALYSIS

Objectives

The primary aim of this study is to determine whether a selective HPE policy following appendicectomy and cholecystectomy for presumed benign diseases is oncologically safe. In addition, we will quantify the potential cost savings of selective HPE.

Study design

This is a Dutch multicentre prospective observational study, registered with ClinicalTrials.gov on the 27th of April 2018 (trial identification number NCT03510923). The study was designed in accordance with the principles of collaborative resident-led Snapshot research. This type of research is primarily led and conducted by surgical residents, supervised by consultants. Snapshot research is particularly suited to investigate a common condition or treatment. By generating a population-based overview, this design rapidly provides insight in current clinical practice. Previous Snapshot studies performed by the Dutch Snapshot Research Group on appendicitis, rectal cancer, and acute left-sided obstructive colon cancer demonstrated that many data can be collected in a short period of time.(17-19)

In the FANCY study, appendices and gallbladders will be systematically assessed by the operating surgeon (or surgical resident) for macroscopic abnormalities suspicious for malignant neoplasms. After visual inspection and digital palpation of the removed specimen, the operating surgeon will report on a predefined scoring form whether macroscopic abnormalities suspicious for a malignant neoplasm are present, and if he or she believes additional microscopic examination by the pathologist is indicated (Supplementary material 1). In case of suspicious macroscopic abnormalities, the surgeon is asked to describe these on the same form. Similarly, the surgeon is requested to specify the indication for HPE, if present. Regardless of the surgeon's assessment, all specimens will be sent for HPE. In this way, routine HPE can be compared to a hypothetical situation of selective HPE.

The FANCY study will be performed in sixty out of seventy-four Dutch hospitals, including academic (n=6), teaching (n=37), and non-teaching hospitals (n=17). Centres that have already implemented a selective HPE policy for gallbladder specimens may choose to either only participate in the appendix part of the FANCY study or to return to the routine policy for the duration of the study period. Hospitals will be allowed to start patient accrual after local approval has been obtained, and a site initiation visit has taken place, including a presentation of the study protocol and instructions for the macroscopic assessment of the specimens. Due to variation in completion of hospitals' local approval procedures, it was decided to open study sites in phases. Every first day of a new month, a group of hospitals will start patient accrual. To avoid bias, all hospitals will include patients for a duration of nine months, even if this means that the required sample size is exceeded.

Study population

Patients of all ages scheduled to undergo appendicectomy for appendicitis or cholecystectomy for cholecystitis or gallstone disease in the elective or non-elective setting will be included.

For peer review only

A potential subject who meets any of the following criteria will be excluded:

Appendix

- Primary indication for surgery: strong clinical/radiological suspicion or histopathological proof of an appendiceal neoplasm;
- Appendices removed as part of more extensive surgery, so-called incidental appendicectomies (e.g. right colectomy);
- Patients included in the ACCURE trial.(20)

Gallbladder

- Primary indication for surgery: strong clinical/radiological suspicion or histopathological proof of GBC;
- Gallbladder removed as part of more extensive surgery, so-called incidental cholecystectomies (e.g. Whipple procedure);
- The presence of a polyp of >10 mm on preoperative imaging.

Study observations

Macroscopic assessment

During the site initiation visits, all steps of a systematically performed macroscopic assessment will be discussed. Instruction videos showing how to perform the macroscopic assessment will be available on the study website during the entire study period.

Surgeon's macroscopic assessment of the appendix

The examination starts with inspection of the appendix and mesoappendix, followed by digital palpation. In consultation with the pathologist, it was decided that opening the appendix is prohibited, since this might impede proper HPE.

Surgeon's macroscopic assessment of the gallbladder

First, the outer surface of the gallbladder is inspected. Then, the gallbladder is incised along its longitudinal axis on the peritoneal side, leaving the cystic duct intact. After removal of stones and bile, the gallbladder mucosa is inspected and palpated. Examples of macroscopic abnormalities are masses, polyps, ulcers, cysts, hardening, irregularity and wall thickening.

HPE

All specimens will be sent to the department of pathology for macro- and microscopic assessment. HPE will be conducted according to the local protocol of the pathology department where the specimen is assessed. In general, this includes macroscopic assessment of the complete specimen, followed by microscopic assessment of samples taken from macroscopic abnormalities and the top (in case of appendices) or cystic duct margin (in case of gallbladders). If no macroscopic abnormalities are present, random samples are taken for microscopic assessment. The latest histological tumour–node–metastasis classification (TNM) of the American Joint Committee on Cancer (AJCC) applicable at the time HPE was performed was used for staging malignancies.(21)

For peer review only

Additional treatment

In case of a histopathologically proven appendiceal neoplasm or GBC, the postoperative management will be discussed in a local multidisciplinary team meeting. If it is decided that an additional resection is required, the specimens of the re-resection will be evaluated for the presence of residual tumour and positive lymph nodes, according to the local protocol.

Outcomes

All outcomes will be analysed separately for appendiceal and gallbladder specimens.

Primary outcomes

Oncological safety

Oncological safety of selective HPE will be assessed by calculating the number of patients per 1,000 examined appendices or gallbladders with the histopathological diagnosis of an appendiceal neoplasm or GBC with clinical consequences benefitting the patient that would have been missed. In case of an appendiceal neoplasm, the following consequences will be considered beneficial: 1) HPE of the re-resection specimen shows residual tumour and/or positive lymph nodes, 2) treatment with (adjuvant) systemic or local chemotherapy, radiotherapy, immunotherapy, or stem cell transplantation, and 3) palliative treatment for metastases detected during staging procedures. If an additional resection is performed following the diagnosis of an appendiceal neoplasm, and no residual tumour and/or positive lymph nodes are found during HPE, this is considered harmful due to the potential risks of surgery the patient is exposed to. For GBC, evidence on the prognostic impact of revisional surgery and adjuvant therapies is limited and inconclusive.(22) For pragmatic reasons, it was decided that all cases of GBC requiring additional diagnostic or therapeutic procedures (i.e. ≥ T1b GBC) will be considered beneficial.

To determine the number of missed diagnoses, only the specimens that would not have been sent for HPE in case of a selective policy (i.e. specimens without an indication for HPE according to the surgeon), will be analysed. In general, it is difficult to determine what incidence of missed neoplasms is acceptable to omit routine HPE. The cut off value for safety of selective HPE was chosen based on data from the Dutch national screening program for colorectal cancer (CRC). The incidence of CRC in asymptomatic patients is 0.8%, and the sensitivity of the immunochemical fecal occult blood test (iFOBT) ranges from 65% to 80%, depending on the number of screenings.(23) As a result, the diagnosis of CRC is missed in 1.6 to 2.8 per 1,000 patients. Since selective HPE implies cost savings, a reduced workload for pathologists, and less risk of overtreatment, a higher incidence of missed diagnoses is acceptable. Therefore, it was decided that selective HPE of appendices and gallbladders will be considered oncologically safe if the number of patients with a neoplasm with clinical consequences benefitting the patient that would have been missed is below 3 per 1,000 examined specimens (approximately twice the incidence of missed CRC in the screening program).

Cost analysis

The economic evaluation will be performed as a cost-minimisation analysis. In addition, a budget impact analysis of selective HPE will be performed from governmental, insurer, and hospital provider perspectives.

For peer review only

Secondary outcomes

1. The incidence of different histopathological diagnoses following appendicectomy and cholecystectomy;
2. Value of the intraoperative assessment (i.e. inspection and palpation) performed by the surgeon for detection of appendiceal neoplasms or GBC;
    a. Incidence of specimens with a recognised appendiceal neoplasm or GBC;
    b. Incidence of specimens with an unrecognised appendiceal neoplasm or GBC;
3. Indication for additional diagnostic or therapeutic procedures following histopathological diagnosis of appendiceal neoplasms or GBC and its clinical consequences, both in terms of benefit and harm;
    a. Incidence of appendiceal neoplasms and GBC requiring additional diagnostic or therapeutic procedures;
    b. Incidence of residual tumour and/or positive lymph nodes found in the re-resection specimen;
    c. Incidence of postoperative complications within 90 days after additional resection.
4. Value of the intraoperative assessment (i.e. inspection and palpation) performed by the surgeon for detection of aberrant findings other than appendiceal neoplasms and GBC.

    Appendiceal specimens

    - Incidence of parasite infection, endometriosis, granulomatosis, and other aberrant findings that would and would not have been sent for HPE.

    Gallbladder specimens

    - Incidence of adenoma, biliary intra-epithelial neoplasm, cholesterol polyp, inflammatory/hyperplastic polyp, adenomyomatosis, and other aberrant findings that would and would not have been sent for HPE.

Group size calculation

Group size calculation is based on the number of appendices and gallbladders with a tumour with clinical consequences benefitting the patient that would have been missed in case of a selective policy. According to systematic reviews, the incidences of appendiceal neoplasms and GBC are 7 per 1,000 and 4 per 1,000 patients, respectively.(1, 2) Data regarding the ability of surgeons to recognise these abnormalities and the consequences of these neoplasms is insufficient. It is however estimated that less than 1 out of 1,000 examined specimens will contain an appendiceal neoplasm or GBC with clinical consequences benefitting the patient that is not recognised by the surgeon during the macroscopic assessment. Selective HPE will be considered safe if this number does not increase to 3 per 1,000 patients. To demonstrate non-inferiority of selective compared to routine HPE, a sample size of 4,462 per cohort achieves a 84% power to detect a difference of 0.002 using a one-sided binomial test at a target significance level of 0.025, assuming a baseline and actual proportion of 0.001, and a non-inferiority limit of 0.00299. The actual significance level achieved by the Fisher's exact test is 0.021. These two cohorts (one for appendices, one for gallbladders) only include the specimens that would not have been sent for HPE in case of a selective policy. If the rate of HPE can be reduced to 20%, 5,578 patients per cohort (4,462 / 0.8) should initially be included.

For peer review only

Study organisation

The FANCY study is coordinated by a PhD candidate (VPB) under supervision of the principle investigator (WAB). The steering committee consists of seven surgeons, of whom two working in academic hospitals (PRR, PJT), and five in teaching hospitals (GJDA, AAWG, KHH, CCR, GDS), a pathologist working in an academic hospital (LK), and a pathologist working in a teaching hospital (VT), three surgical residents (ACK, HAS, JLPV), three PhD candidates (BJGAC, EAJS, JJ), and a clinical methodologist and health economist (MGWD), besides the coordinating PhD candidate and principle investigator. All local principal investigators and the residents, physician assistants and research nurses who are responsible for data collection, will be mentioned in alphabetical order as collaborators on all publications deriving from the FANCY study databases.

Data collection

The local study team of each participating hospital will be responsible for entering the prospectively collected data into an electronic case record form (CRF) build with Castor EDC, which is ISO 27001 and NEN 7510 certified.(24) Pre- and intraoperative data will be processed after surgery, and complemented with the postoperative histopathological outcomes when the pathology report is available (± two weeks after surgery). Pre- and postoperative data will be obtained from the electronic patient database (EPD) and pathology reports. Intraoperative data will be obtained from the scoring forms that will be filled in by the surgeon after examination of the specimen. In case a neoplasm is found during HPE, additional data about postoperative management, including details of additional diagnostic tests and/or treatment, postoperative morbidity and HPE of re-resection specimens, will be collected.

Monitoring of the primary endpoint

The reliability and quality of the primary endpoint will be assured in three ways: 1) revision of all pathology reports, 2) source data verification by remote monitoring of all cases with a histopathological diagnosis of appendiceal neoplasm or GBC, and 3) estimation of the incidence of appendiceal neoplasms and GBC in the group of eligible patients that were unintentionally not included.

Revision of the pathology reports

Under supervision of the two pathologists of the steering committee (LK, VT), the coordinating investigator (VPB) will revise all pathology reports. All histopathological diagnoses will be assigned to one of the predefined categories, as shown in Table 1.

Table 1. Histopathological diagnoses after appendicectomy and cholecystectomy.

APPENDICES	GALLBLADDERS
Normal appendix	Normal gallbladder
Acute inflammation	Acute inflammation
Chronic inflammation and reactive changes	Chronic inflammation and reactive changes

For peer review only

Appendiceal neoplasms	Gallbladder neoplasms
Neuro-endocrine neoplasm	Adenoma
Non-invasive epithelial neoplasm	Biliary intraepithelial neoplasm
Invasive epithelial neoplasm	Carcinoma
Lymphoma	Other malignant neoplasms
**Non-neoplastic aberrant findings**	**Non-neoplastic aberrant findings**
Parasitic infection	Cholesterol polyp
Endometriosis	Inflammatory / hyperplastic polyp
Granulomatous disease	Adenomyomatosis
Other	Other

Source data verification

Independent remote monitoring will be performed by a qualified monitor of the Clinical Research Unit of the Amsterdam UMC. Monitoring will be limited to all cases with a histopathological diagnosis of an appendiceal neoplasm or GBC. The quality assessment will focus on comparing entered data with source documents. Since no informed consent is obtained in this study, anonymised source documents of relevant patients will be supplied by the local study teams.

Estimation of the incidence of neoplasms in unintentionally not included patients

Since the macroscopic assessment of appendices and gallbladders is currently not routine practice, surgeons and residents might unintentionally forget to assess the specimen and fill in the scoring form. It is expected that the macroscopic assessment will not be performed in approximately 5-10% of all eligible patients. In order to determine whether our patient cohort is representative for all patients undergoing an appendicectomy or cholecystectomy, the incidence of appendiceal neoplasms and GBC in the group of patients that were not included has to be determined. This will be done in collaboration with PALGA, the Dutch nationwide network and registry of histopathology and cytopathology that contains pathology reports of all pathology laboratories in the Netherlands with complete coverage of reports since 1991.(25) The PALGA database will be used to assess the number of patients in the participating centres that were unintentionally not included in the FANCY study. By means of comparing the total number of appendiceal neoplasms and GBC found in the PALGA database to the study database, we will be able to identify the number of patients that were not registered in the FANCY study. In collaboration with a staff member of PALGA, the individual pathology reports (without patient identifying information) of these patients will be checked for exclusion criteria. Consequently, the (estimated) incidence of appendiceal neoplasms and GBC in the group of unintentionally not included patients will be known.

Cleaning and locking of the database

The database will be locked and exported for statistical analysis as soon as all data is entered, and all missing items are checked with the local study team. After locking, the database will be archived in a licensed repository.

For peer review only

Predefined statistical analysis plan

General principles

The analyses will be performed after data entry is completed, monitoring and cleaning of the data has been performed, and the statistical analysis plan is accepted for publication. For the primary analyses, all patients who underwent an appendicectomy for appendicitis or cholecystectomy for cholecystitis or gallstone disease will be included. All analyses described below will be performed using the latest version of SPSS statistics (IBM Corp., Armonk, NY, USA) at the time of analysis.

Baseline characteristics

Baseline characteristics will be expressed as medians and interquartile ranges (IQR), or counts and percentages. Baseline characteristics will be presented as shown in Table 2 (appendices) and Table 3 (gallbladders).

Table 2. Baseline characteristics (appendices).

		**Total (n=)**
Age, years		Median (IQR)
Sex, n (%)	Female	n (% of 'Total')
	Male	n (% of 'Total')
Preoperative imaging, n (%)	Ultrasound	n (% of 'Total')
	Ultrasound + CT	n (% of 'Total')
	Ultrasound + MRI	n (% of 'Total')
	Ultrasound + CT + MRI	n (% of 'Total')
	CT	n (% of 'Total')
	MRI	n (% of 'Total')
	CT + MRI	n (% of 'Total')
	No preoperative imaging	n (% of 'Total')
Hospital	Academic hospital	n (% of 'Total')
	Teaching hospital	n (% of 'Total')
	Non-teaching hospital	n (% of 'Total')
Macroscopic assessment performed by	Surgeon	n (% of 'Total')
	Resident	n (% of 'Total')
	Both	n (% of 'Total')

CT, computed tomography; IQR, interquartile range; MRI, magnetic resonance imaging

For peer review only

Table 3. Baseline characteristics (gallbladder).

		Total (n=)
Age, years		Median (IQR)
Sex, n (%)	Female	n (% of 'Total')
	Male	n (% of 'Total')
Preoperative diagnosis, n (%)	Cholecystitis	n (% of 'Total')
	Symptomatic cholelithiasis	n (% of 'Total')
Preoperative imaging, n (%)	Ultrasound	n (% of 'Total')
	Ultrasound + CT	n (% of 'Total')
	Ultrasound + MRI	n (% of 'Total')
	Ultrasound + CT + MRI	n (% of 'Total')
	CT	n (% of 'Total')
	MRI	n (% of 'Total')
	CT + MRI	n (% of 'Total')
	Other	n (% of 'Total')
	No preoperative imaging	n (% of 'Total')
Surgical setting	Acute	n (% of 'Total')
	Elective	n (% of 'Total')
Hospital	Academic hospital	n (% of 'Total')
	Teaching hospital	n (% of 'Total')
	Non-teaching hospital	n (% of 'Total')
Macroscopic assessment performed by	Surgeon	n (% of 'Total')
	Resident	n (% of 'Total')
	Both	n (% of 'Total')

CT, computed tomography; IQR, interquartile range; MRI, magnetic resonance imaging

For peer review only

Primary outcomes

Oncological safety

The number of patients with a histopathological diagnosis of an appendiceal neoplasm or GBC with clinical consequences benefitting the patient will be reported in absolute numbers and percentages, and as number per 1,000 examined specimens for both strategies of HPE (routine and selective). The data will be presented as in Figure 1 (appendices) and Figure 2 (gallbladders). For analysis of the primary outcome, only the specimens that would not have been sent for HPE in case of a selective policy (i.e. specimens without an indication for HPE according to the surgeon), will be analysed. A selective policy will be considered safe, if, following an exact test, the one-sided upper limit at a 97.5% confidence level of the proportion of missed malignancies falls below 3 per 1,000 examined specimens. The influence of the assessor of the specimen (surgeon vs. resident) and hospital (academic hospital vs. teaching hospital vs. non-teaching hospital) on the primary outcome will be assessed with Poisson regression.

Costs

1) Economic evaluation

Considering that – under the non-inferiority hypothesis – 1 or 2 per 1,000 patients at maximum will experience health consequences from routine HPE, and 3 to 4 per 1,000 patients may experience transient harm following unnecessary additional treatment, differences in effectiveness between the routine and selective strategy can best

be addressed qualitatively (e.g. by case reports). Quantitatively, a cost analysis from a health care provider will be the main focus of research.

During the study, all specimens will be sent for HPE, so the selective policy will not be observed. The comparison between both strategies will therefore be done through decision tree analysis. Observed distributions of histopathological findings under the routine policy will be used to define chance nodes in the reference tree of the model. Alternatively, both the distribution of histopathological findings in specimens that would have been sent for HPE as the distribution of histopathological findings in specimen that would not have been sent for HPE in case of a selective policy will be used to define chance nodes in the other main model tree.

In addition to the initial HPE, costs of other resources (e.g. additional treatment, additional HPE, hospital stay) will be assigned to each end node in the model. If costs were observed under routine examination, but the specimen would not have been sent for HPE in case of a selective policy, they will be ignored if related to unnecessary use of health care resources. However, if these costs were justified, and the specimen would not have been sent for HPE in case of a selective policy, it will be assumed that these costs would nevertheless be generated at a later stage during the disease course, and thus included in the decision tree. In addition, a scenario analysis will be run with a 50% surplus penalty of these costs to compensate for yet unobserved extra costs of delayed health care at a later disease stage. Unit costing of hospital resources will be based on the Erasmus University Rotterdam / National Health Care Institute guideline for costing in health care research.(26) If specific unit costs are lacking in the guideline, local bottom-up or top-down costing initiatives in participating hospitals (e.g. Amsterdam UMC) will be used.

All probabilities at the chance nodes will be assumed Beta-distributed. Multiple theoretical distributions will be assessed for fitting the (observed) distributions of health care costs at the end nodes of the decision tree. If theoretical fits seem insufficient, a uniform distribution will be defined for the (observed) cost data. Monte Carlo simulation will be applied based on 25,000 draws from each distribution of input parameters. It is expected that a time horizon for the cost analysis of six months is sufficient to reliably estimate the cost difference between routine and selective HPE. Separate models will be built for the analyses of appendices and gallbladders.

2) Budget Impact Analysis

The budget impact analysis of selective HPE will be performed from governmental, insurance, and hospital provider perspectives for a four-year budget period, starting with the first full budget year after completion of the trial. The budget impact will be expressed in millions of euros. Primarily, the budget for care by medical specialists (code 0303), such as pathologists and surgeons, will be affected. For all perspectives, the reimbursement guidelines from the Dutch Healthcare Authority will be applied to the estimate actual expenses.

In case of negotiable reimbursement levels, the 10% trimmed mean purchase price per unit as provided by www.opendisdata.nl (e.g. DBC-code 119599010) will be used. Data on the incidences of performed appendicectomies and cholecystectomies will be gathered from public data sources (www.opendisdata.nl;

For peer review only

www.statline.cbs.nl), and linearly extrapolated to forecast the numbers during the period for budget impact analysis.

Secondary outcomes

All incidences of histopathological diagnoses will be reported in absolute numbers and percentages. The incidence of different histopathological diagnoses will be presented as shown in Table 4 (appendices) and Table 5 (gallbladders). These tables will also provide information on which specimens would and would not have been sent for HPE in case of a selective policy. Table 6 will show whether or not specimens containing an appendiceal neoplasm or GBC were reported as suspicious by the surgeon and whether or not the surgeon believed HPE was indicated. Details on all patients with an appendiceal neoplasm or GBC will be presented as proposed in Table 7 (appendices) and Table 8 (gallbladders).

Table 4. Histopathological diagnoses after appendicectomy for appendicitis.

			**Total (n=)**	**Indication for HPE[a] (n=)**	**No indication for HPE[a] (n=)**
Histopathological diagnosis		Normal appendix	n (% of 'Total)	n	n
		Acute inflammation	n (% of 'Total')	n[b]	n[c]
		Chronic inflammation and reactive changes	n (% of 'Total')	n	n
	Appendiceal neoplasms	Neuro-endocrine neoplasm	n (% of 'Total')	n	n
		Non-invasive epithelial neoplasm	n (% of 'Total')	n	n
		Invasive epithelial neoplasm	n (% of 'Total')	n	n
		Lymphoma	n (% of 'Total')	n	n
	Non-neoplastic aberrant findings	Parasitic infection	n (% of 'Total')	n	n
		Endometriosis	n (% of 'Total')	n	n
		Granulomatous disease	n (% of 'Total')	n	n
		Other	n (% of 'Total')	n	n

HPE, histopathological examination

a. According to the operating surgeon or surgical resident.
b. Uncomplicated acute appendicitis (n=), complicated acute appendicitis (n=); as reported in pathology report.
c. Uncomplicated acute appendicitis (n=), complicated acute appendicitis (n=); as reported in pathology report.

For peer review only

Table 5. Histopathological diagnoses after cholecystectomy for presumed benign gallbladder diseases.

			Total (n=)	Indication for HPE[a] (n=)	No indication for HPE[a] (n=)
Histopathological diagnosis		Normal gallbladder	n (% of 'Total)	n	n
		Acute inflammation	n (% of 'Total')	n	n
		Chronic inflammation and reactive changes	n (% of 'Total')	n	n
	Gallbladder neoplasms	Adenoma	n (% of 'Total')	n	n
		Biliary intraepithelial neoplasm	n (% of 'Total')	n	n
		Carcinoma	n (% of 'Total')	n	n
		Other malignant neoplasms	n (% of 'Total')	n[b]	n[c]
	Non-neoplastic aberrant findings	Cholesterol polyp	n (% of 'Total')	n	n
		Inflammatory / hyperplastic polyp	n (% of 'Total')	n	n
		Adenomyomatosis	n (% of 'Total')	n	n
		Other	n (% of 'Total')	n	n

HPE, histopathological examination

a. According to the operating surgeon or surgical resident.
b. Details on histology.
c. Details on histology.

For peer review only

Table 6. Value of the intraoperative assessment by the surgeon for detection of appendiceal neoplasms / GBC

	Appendiceal neoplasm / GBC (n=)	No appendiceal neoplasm / GBC (n=)	Total (n=)
**Presence of abnormalities suspicious for malignancy**	n (% of 'Appendiceal neoplasm / GBC)	n (% of 'No appendiceal neoplasm / GBC)	n (% of 'Total')
**Indication for HPE**	n (% of 'Appendiceal neoplasm / GBC)	n (% of ' No appendiceal neoplasm / GBC)	n (% of 'Total')

GBC, gallbladder cancer; HPE, histopathological examination

Table 7. Details of patients with a histopathological diagnosis of an appendiceal neoplasm.

Case	Sex, Age	Preoperative imaging	Assessor	Macroscopic abnormalities suspicious for neoplasm	Indication for HPE according to surgeon	Histopathological diagnosis	Additional diagnostic and/or therapeutic procedures	Remaining tumour tissue	Positive lymph nodes	90-day complications
1										
2										
3										
…										

Table 8. Details of patients with a histopathological diagnosis of GBC.

Case	Sex, Age	Preoperative imaging	Preoperative diagnosis	Surgical setting	Assessor	Macroscopic abnormalities suspicious for neoplasm	Indication for HPE according to surgeon	Histopathological diagnosis	Additional diagnostic and/or therapeutic procedures	Residual disease	90-day complications
1											
2											
3											
…											

Several exploratory subgroup analyses will be performed. For appendiceal specimens, the influence of age (adults vs. children) on the incidence of different histopathological diagnoses will be evaluated and reported in a similar way as shown in Table 4. For gallbladder specimens, a subgroup analysis on the influence of preoperative diagnosis (cholecystitis vs. gallstone disease) on the incidence of different histopathological diagnoses will be performed. Furthermore, the influence of the assessor (surgeon vs. resident) and hospital (academic hospital vs. teaching hospital vs. non-teaching hospital) on the rate of specimens that would have been sent for HPE will be reported.

Current status of the study

The study was registered with ClinicalTrials.gov on the 27th of April 2018 (trial identification number NCT03510923) and in the Netherlands Trial Register on the 16th of April 2018 under number NTR7151 (www.trialregister.nl). Recruitment of patients started in May 2018. At time of submission, November 2019, 53 of 60 hospitals have finished the 13 months period of data collection (nine months accrual followed by four months for data entry) and 6,902 and 8,387 patients have been included in the appendices and gallbladders databases, respectively.

Manuscripts and authorship

The steering committee of the FANCY study will share the results irrespective of the outcomes. The outcomes as described in this protocol will be reported in two manuscripts, one for the appendices and one for the gallbladders. These manuscripts will be submitted with the steering committee as co-authors and all other investigators as collaborators. The coordinating investigator (VPB) and principal investigator (WAB) will be first and senior author

For peer review only

on both manuscripts, respectively. If the results of the economic evaluation are reported separately, senior authorship for this manuscript will be shared by WAB and MGWD. For the appendices manuscript, the other PhD candidates will be second (JJ), third (BJGAC) and fourth author (EAJS). If possible, JLPV, EAJS and BJAGC will share second authorship on the gallbladder manuscript. The other members of the steering committee will be co-authors on both publications. All local principal investigators and the residents, physician assistants and research nurses who were responsible for data collection, will be mentioned in alphabetical order as collaborators. All efforts will be made to link the collaborators to the final publications in indexed databases.

Patient and public involvement

Patients and public were not involved in designing the study.

For peer review only

ETHICS AND DISSEMINATION

Ethical aspects and informed consent

This study will be performed in accordance with the principles of Good Clinical Practice, the Dutch Agreement on Medical Treatment Act (WGBO) and the European General Data Protection Regulation. The study protocol was reviewed by the Institutional Review Board of the Amsterdam UMC, location AMC, which decided that the Dutch Medical Research Involving Human Subjects Act (WMO) is not applicable. In all participating centres, approval for execution of the FANCY study has been obtained from the local Institutional Review Board before the start of inclusion of patients.

In the FANCY study, a large number of patients will be included in a relatively short period of time. After consultation with the legal department of the Amsterdam UMC, it was decided that no written informed consent will be requested for the use of patients' data. Obtaining written informed consent of all included patients during the usually short hospital admission would be futile and impede the execution of this study. Participation in the FANCY study does not have any treatment consequences for patients, as there is no change in current clinical practice. Patients will easily postpone their decision on participation. Moreover, it was suggested that certain patient groups (e.g. young patients, patients with a complicated postoperative course, patients with histopathological findings requiring additional hospital visits) tend to provide informed consent more often, which would introduce selection bias. A deferred consent procedure including a phone call in the postoperative period was considered but deemed impractical due to the large number of health care providers involved. For these reasons, it was decided that the extensive effort to obtain informed consent does not compete with the relatively small amount of non-identifiable data that is collected in the FANCY study. Alternatively, patients will be offered the opportunity to refuse the use of their data by using an opt-out procedure. All patients that underwent an appendicectomy or cholecystectomy will receive a leaflet with brief information about the study. It will be explained that all data will be extracted from the patient's charts followed by deidentification and no additional investigations are required. When a patient or its relatives object to participate, the patient will be excluded from the study and data will not be entered into the database.

Dissemination

During the study, all collaborators will be updated about the progress of the study by monthly newsletters. The results of the FANCY study will be presented at national and international conferences and submitted for publication in an international peer-reviewed scientific journal. The Dutch Surgical Society (NVVH), which is responsible for revision of the guidelines, recognises the relevance of this research and supports the implementation of the results. As secretary of the Board of Directors of the NVVH (GJDA) and chairman of the guideline committee 'Appendicitis' (CCR), two of our steering committee members are involved in the revision of the guidelines, which ensures that the guidelines will be adjusted according to the results of the FANCY study.

For peer review only

ACKNOWLEDGEMENTS

None.

AUTHOR CONTRIBUTIONS

VPB, MGWD and WAB have made substantial contributions to the conception and design of this study and have been involved in drafting the manuscript or revising it critically for important intellectual content. BJGA have made substantial contributions to the design of this study and have been involved in drafting the manuscript. EAJS, JJ, ACK, HAS, JLPV, GJDA, AAWG, KHH, LK, PRR, CCR, GDS, PJT and VT have made substantial contributions to the design of this study and the organisation of this trial and have revised the manuscript critically for important intellectual content. All authors have given final approval of the version to be published.

FUNDING

This investigator initiated study was supported by the Netherlands Organisation for Health Research and Development (ZonMw) grant number 843002822, of which the Dutch Ministry of Health, Welfare and Sports and the Dutch Organisation for Scientific Research are the main commissioning organisations. ZonMw has not played a role in designing the study and data collection, nor will it influence the analysis and writing process of the manuscripts.

COMPETING INTERESTS

None declared.

PATIENT CONSENT FOR PUBLICATION

Not required.

For peer review only

REFERENCES

1. Swank HA, Eshuis EJ, Ubbink DT, et al. Is routine histopathological examination of appendectomy specimens useful? A systematic review of the literature. *Colorectal Dis*. 2011;13(11):1214-21.
2. Swank HA, Mulder IM, Hop WC, et al. Routine histopathology for carcinoma in cholecystectomy specimens not evidence based: a systematic review. *Surg Endosc*. 2013;27(12):4439-48.
3. Khan RA, Ghani I, Chana RS. Routine histopathological examination of appendectomy specimens in children: is there any rationale? *Pediatr Surg Int*. 2011;27(12):1313-5.
4. Jahan B, Najeeb S, Shaikh AW. Acute appendicitis, correlating histopathological findings with clinical - is histopathology needed for all? *PJMHS*. 2016;10(1):118-21.
5. Van Vliet JLP, Van Gulik TM, Verbeek PCM. Is it necessary to send gallbladder specimens for routine histopathological examination after cholecystectomy? The use of macroscopic examination. *Digestive Surgery*. 2014;30(4-6):472-5.
6. Emmett CD, Barrett P, Gilliam AD, et al. Routine versus selective histological examination after cholecystectomy to exclude incidental gallbladder carcinoma. *Ann R Coll Surg Engl*. 2015;97(7):526-9.
7. Olthof PB, Metman MJH, de Krijger RR, et al. Routine Pathology and Postoperative Follow-Up are Not Cost-Effective in Cholecystectomy for Benign Gallbladder Disease. *World J Surg*. 2018;42(10):3165-70.
8. Deng YL, Xiong XZ, Zhou Y, et al. Selective histology of cholecystectomy specimens--is it justified? *J Surg Res*. 2015;193(1):196-201.
9. Pape UF, Niederle B, Costa F, et al. ENETS Consensus Guidelines for Neuroendocrine Neoplasms of the Appendix (Excluding Goblet Cell Carcinomas). *Neuroendocrinology*. 2016;103(2):144-52.
10. Aloia TA, Jarufe N, Javle M, et al. Gallbladder cancer: expert consensus statement. *HPB (Oxford)*. 2015;17(8):681-90.
11. Romero-Gonzalez RJ, Garza-Flores A, Martinez-PerezMaldonado L, et al. Gallbladder selection for histopathological analysis based on a simple method: a prospective comparative study. *Annals of the Royal College of Surgeons of England*. 2012;94(3):159-64.
12. Tayeb M, Rauf F, Ahmad K, et al. Is it necessary to submit grossly normal looking gall bladder specimens for histopathological examination? *Asian Pac J Cancer Prev*. 2015;16(4):1535-8.
13. Corten B, Alexander S, van Zwam PH, et al. Outcome of Surgical Inspection of the Gallbladder in Relation to Final Pathology. *J Gastrointest Surg*. 2019;23(6):1130-4.
14. NVVH. Evidence-based richtlijn. Nederlandse Vereniging voor Heelkunde, Acute appendicitis. 2019.
15. NVVH. Evidence-based richtlijn. Nederlandse Vereniging voor Heelkunde, Galsteenlijden. 2016.
16. Corten B, Leclercq WKG, Dejong CH, et al. Selective Histological Examination After Cholecystectomy: An Analysis of Current Daily Practice in The Netherlands. *World J Surg*. 2019.
17. van Rossem CC, Bolmers MD, Schreinemacher MH, et al. Prospective nationwide outcome audit of surgery for suspected acute appendicitis. *Br J Surg*. 2016;103(1):144-51.
18. Borstlap WAA, Westerduin E, Aukema TS, et al. Anastomotic Leakage and Chronic Presacral Sinus Formation After Low Anterior Resection: Results From a Large Cross-sectional Study. *Ann Surg*. 2017;266(5):870-7.
19. Amelung FJ, Borstlap WAA, Consten ECJ, et al. Propensity score-matched analysis of oncological outcome between stent as bridge to surgery and emergency resection in patients with malignant left-sided colonic obstruction. *Br J Surg*. 2019;106(8):1075-86.

For peer review only

20. Gardenbroek TJ, Pinkney TD, Sahami S, et al. The ACCURE-trial: the effect of appendectomy on the clinical course of ulcerative colitis, a randomised international multicenter trial (NTR2883) and the ACCURE-UK trial: a randomised external pilot trial (ISRCTN56523019). *BMC Surg*. 2015;15:30.
21. Amin MB, Edge S, Greene F, et al. AJCC Cancer Staging Manual (8th edition). 2017.
22. Soreide K, Guest RV, Harrison EM, et al. Systematic review of management of incidental gallbladder cancer after cholecystectomy. *Br J Surg*. 2019;106(1):32-45.
23. Wiersma T, de Wit N, Drenthen T. NHG-Standpunt Bevolkingsonderzoek darmkanker. 2013.
24. Castor EDC. Castor Electronic Data Capture 2019 [27 Aug. 2019]. Available from: https://castoredc.com.
25. Casparie M, Tiebosch AT, Burger G, et al. Pathology databanking and biobanking in The Netherlands, a central role for PALGA, the nationwide histopathology and cytopathology data network and archive. *Cell Oncol*. 2007;29(1):19-24.
26. Zorginstituut N. Richtlijn voor het uitvoeren van economische evaluaties in de gezondheidszorg. Diemen. 2015.

For peer review only

FIGURE LEGENDS

Figure 1. Number of patients with an appendiceal neoplasm with clinical consequences benefitting the patient that would have been diagnosed (green box) and missed (red box) in case of a selective policy. The gray dotted line indicates the total number of patients benefitting from clinical consequences of an appendiceal neoplasm, that would have been diagnosed in case of a routine policy.

Figure 2. Number of patients with gallbladder cancer with clinical consequences benefitting the patient that would have been diagnosed (green box) and missed (red box) in case of a selective policy. The gray dotted line indicates the total number of patients benefitting from clinical consequences of gallbladder cancer, that would have been diagnosed in case of a routine policy.

For peer review only

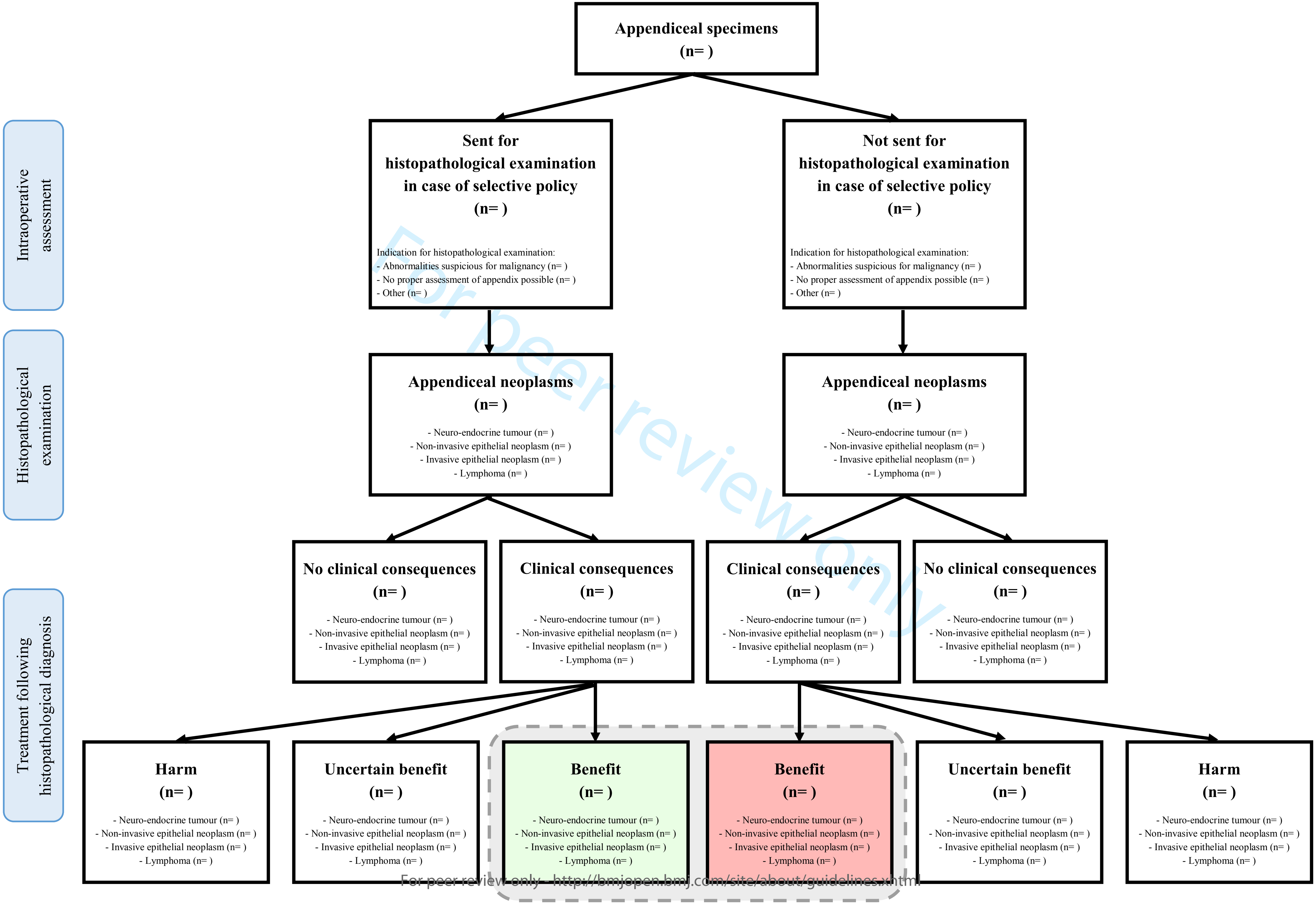

For peer review only

For peer review only - http://bmjopen.bmj.com/site/about/guidelines.xhtml

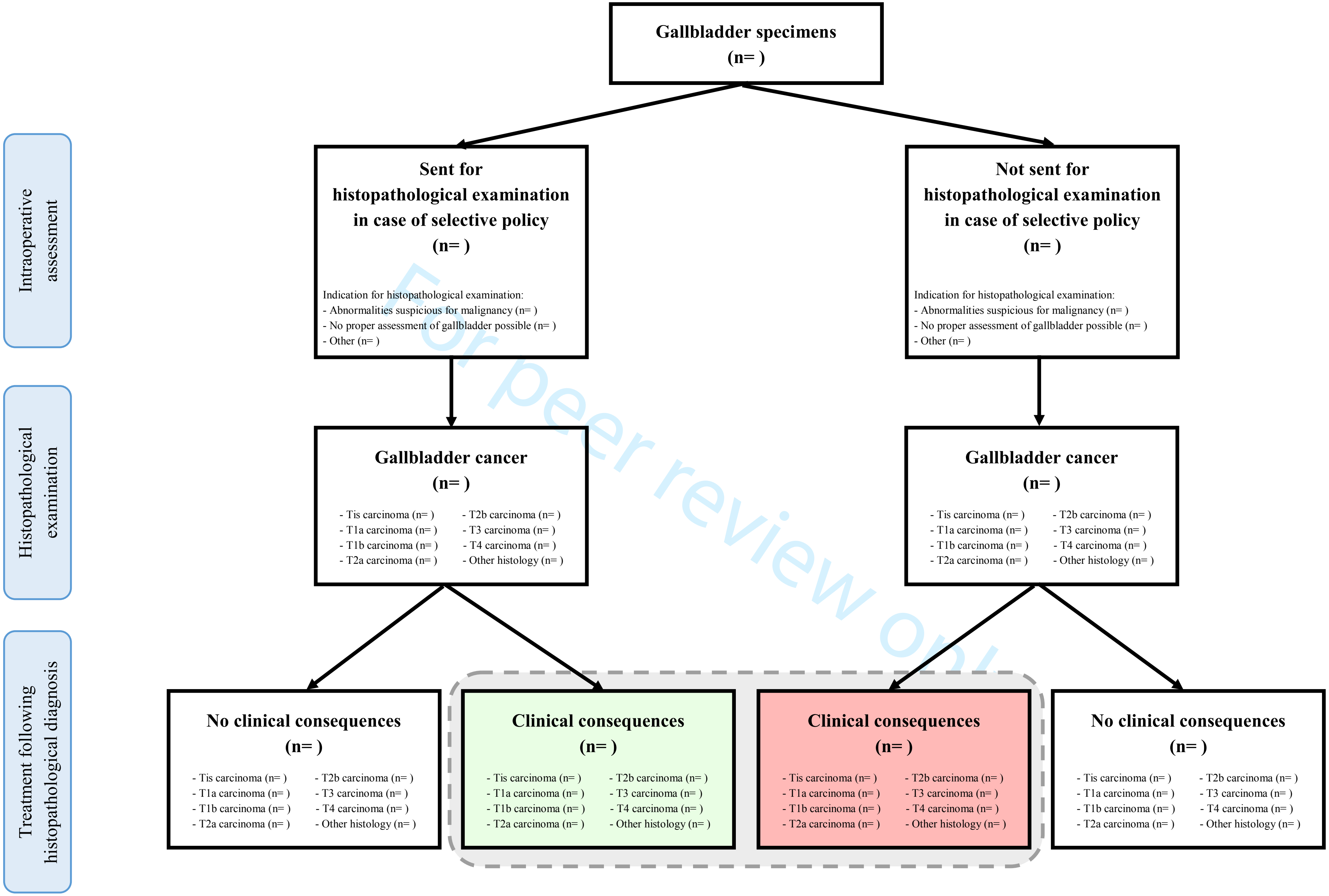

For peer review only - http://bmjopen.bmj.com/site/about/guidelines.xhtml

SUPPLEMENTARY MATERIAL 1

Questions of the scoring form 'Macroscopic examination of the appendix'

1) Who performed the macroscopic examination of the appendix?
Multiple answers possible
O Surgeon
O Surgical resident

2) Are there any macroscopic abnormalities suspicious for a malignant neoplasm during visual inspection and/or digital palpation?
O Yes (please specify below)
O No
O Proper assessment is not possible due to inflammation

2a. If yes, specify the macroscopic abnormalities
Multiple answers possible
O Visible tumour
O Palpable tumour
O Other, namely …

3) Do you believe additional histopathological examination by the pathologist is indicated?
O Yes, because of the above-mentioned abnormalities.
O Yes, because proper assessment of the appendix was not possible.
O Yes, because …
O No

Questions of the scoring form 'Macroscopic examination of the gallbladder'

1) Who performed the macroscopic examination of the gallbladder?
Multiple answers possible
O Surgeon
O Surgical resident

2) Are there any macroscopic abnormalities suspicious for a malignant neoplasm during visual inspection and/or digital palpation?
O Yes (please specify below)
O No

2a. If yes, specify the macroscopic abnormalities
Multiple answers possible

O Ulcer	O Wall thickening
O Cyst	O Polyp
O Hardening	O Tumour suspicious for malignancy
O Irregularity	O Other, namely …

3) Do you believe additional histopathological examination by the pathologist is indicated?
O Yes, because of the above-mentioned abnormalities.
O Yes, because …
O No

For peer review only
