# Supplementary material for: Safety and cost analysis of selective histopathological examination following appendicectomy and cholecystectomy (FANCY study): protocol and statistical analysis plan of a prospective observational multicentre study
Source: BMJ Open. 2019 Dec 23;9(12):e035912. doi: 10.1136/bmjopen-2019-035912 (PMC7008446; doi:10.1136/bmjopen-2019-035912)
Supplement: Reviewer comments [file bmjopen-2019-035912.reviewer_comments.pdf]

PEER REVIEW HISTORY

BMJ Open publishes all reviews undertaken for accepted manuscripts. Reviewers are asked to complete a checklist review form (**http://bmjopen.bmj.com/site/about/resources/checklist.pdf**) and are provided with free text boxes to elaborate on their assessment. These free text comments are reproduced below.

ARTICLE DETAILS

**TITLE (PROVISIONAL)**	Safety and cost analysis of selective histopathological examination following appendicectomy and cholecystectomy (FANCY study); protocol and statistical analysis plan of a prospective observational multicentre study.
**AUTHORS**	Bastiaenen, Vivian P; Corten, Bartholomeus JGA; de Savornin Lohman, Elise AJ; de Jonge, Joske; Kraima, Anne C; Swank, Hilko A; van Vliet, Jaap LP; van Acker, Gijs JD; van Geloven, Anna AW; in 't Hof, Klaas H; Koens, Lianne; de Reuver, Philip; van Rossem, Charles C; Slooter, Gerrit D; Tanis, Pieter J; Terpstra, Valeska; Dijkgraaf, Marcel GW; Bemelman, Willem A

This article was not externally reviewed at BMJ Open. Protocols that have been independently assessed prior to submission to BMJ Open are usually fast-tracked to publication on the grounds that further substantial changes will not be possible. This independent assessment will usually be external, independent review for both a substantial grant award from a non-commercial or government funder and ethics approval.
